# Unique Features of *Mycobacterium abscessus* Biofilms Formed in Synthetic Cystic Fibrosis Medium

**DOI:** 10.3389/fmicb.2021.743126

**Published:** 2021-10-29

**Authors:** Juan M. Belardinelli, Wei Li, Charlotte Avanzi, Shiva K. Angala, Elena Lian, Crystal J. Wiersma, Zuzana Palčeková, Kevin H. Martin, Bhanupriya Angala, Vinicius C. N. de Moura, Callan Kerns, Victoria Jones, Mercedes Gonzalez-Juarrero, Rebecca M. Davidson, Jerry A. Nick, Bradley R. Borlee, Mary Jackson

**Affiliations:** ^1^Mycobacteria Research Laboratories, Department of Microbiology, Immunology, and Pathology, Colorado State University, Fort Collins, CO, United States; ^2^Department of Microbiology, Immunology, and Pathology, Colorado State University, Fort Collins, CO, United States; ^3^Center for Genes, Environment, and Health, National Jewish Health, Denver, CO, United States; ^4^Department of Medicine, National Jewish Health, Denver, CO, United States; ^5^Department of Medicine, University of Colorado Anschutz Medical Campus, Aurora, CO, United States

**Keywords:** *Mycobacterium abscessus*, biofilm, antibiotic tolerance, polysaccharide, extracellular DNA, lipid

## Abstract

Characterizing *Mycobacterium abscessus* complex (MABSC) biofilms under host-relevant conditions is essential to the design of informed therapeutic strategies targeted to this persistent, drug-tolerant, population of extracellular bacilli. Using synthetic cystic fibrosis medium (SCFM) which we previously reported to closely mimic the conditions encountered by MABSC in actual cystic fibrosis (CF) sputum and a new model of biofilm formation, we show that MABSC biofilms formed under these conditions are substantially different from previously reported biofilms grown in standard laboratory media in terms of their composition, gene expression profile and stress response. Extracellular DNA (eDNA), mannose-and glucose-containing glycans and phospholipids, rather than proteins and mycolic acids, were revealed as key extracellular matrix (ECM) constituents holding clusters of bacilli together. None of the environmental cues previously reported to impact biofilm development had any significant effect on SCFM-grown biofilms, most likely reflecting the fact that SCFM is a nutrient-rich environment in which MABSC finds a variety of ways of coping with stresses. Finally, molecular determinants were identified that may represent attractive new targets for the development of adjunct therapeutics targeting MABSC biofilms in persons with CF.

## Introduction

Over the last 10 years, rapidly growing nontuberculous mycobacteria (NTM) of the *Mycobacterium abscessus* complex (MABSC) have emerged as important human pathogens causing an increasing number of pulmonary infections among cystic fibrosis (CF) and non-CF bronchiectasis patients globally (Floto and Haworth, 2015; Park and Olivier, 2015; Martiniano et al., 2019).

Like other mycobacterial pathogens, MABSC species are intracellular pathogens that primarily infect macrophages but can also target neutrophils, epithelial cells, and endothelial cells (Davidson et al., 2011; Garcia-Perez et al., 2011; Malcolm et al., 2013; Viljoen et al., 2017; Matsuyama et al., 2018). While MABSC strains have long been known to form biofilms in the environment and under laboratory growth conditions (Xiang et al., 2014; Faria et al., 2015; Chakraborty and Kumar, 2019; Chakraborty et al., 2021; Dokic et al., 2021), only recently have studies in CF and non-CF infected lung tissues provided support for the assumption that extracellular MABSC biofilm growth also occurs during human pulmonary infections (Qvist et al., 2015; Fennelly et al., 2016; Hoiby, 2017). The finding of biofilms, herein defined as microcolonies of MABSC embedded within a matrix, in the thickened alveolar walls, airways, and lung cavity of patients has important clinical implications. The presence of biofilms where bacilli in different metabolic states (including intrinsically drug-tolerant semi-dormant cells) persist may help explain why MABSC lung infections are usually incurable with antibiotic therapy alone and why adjunctive surgical resection of cavities may improve treatment outcomes. MABSC biofilms may further promote the colonization of the respiratory epithelium as recently illustrated by studies on *Mycobacterium avium* (Yamazaki et al., 2006; Babrak et al., 2015a, b) or attenuate phagocytic cell (Rose and Bermudez, 2014) and neutrophil functions (Malcolm et al., 2013) to facilitate immune evasion leading to persistent infection (Richards and Ojha, 2014).

Owing to the fact that the most in-depth genetic and biochemical studies on mycobacterial biofilm thus far have been conducted on *Mycobacterium spp*. other than MABSC (e.g., *Mycobacterium tuberculosis*, *M. avium*, *M. chelonae*, *M. smegmatis*, and *M. ulcerans*) and have been performed in defined laboratory media that do not reflect the physical conditions encountered by MABSC in the airway and lung of the infected host, the environmental signals and molecular determinants governing MABSC biofilm development *in vivo* remain, for the most part, unknown (Carter et al., 2003; Marsollier et al., 2007; Ojha and Hatfull, 2007; Ojha et al., 2008; Rose and Bermudez, 2016; Trivedi et al., 2016; Chakraborty and Kumar, 2019; Vega-Dominguez et al., 2020; Dokic et al., 2021). In this study, we phenotypically and genotypically characterized the biofilm formed by a *M. abscessus* subsp. *abscessus* CF isolate in synthetic CF medium which we found to closely mimic the conditions encountered by MABSC in actual CF sputum (Wiersma et al., 2020). We highlight the substantial differences these biofilms display from MABSC biofilms formed in standard laboratory media and reveal new targets for therapeutic intervention directed to this population of persistent, drug-tolerant, extracellular bacilli.

## Materials and Methods

### Strains and Culture Media

*Mycobacterium abscessus* subsp. *abscessus* clinical isolate NJH12 was from a patient with CF at National Jewish Health (Denver, CO, United States). *Mabs* NJH12 was grown under agitation at 37°C in Middlebrook 7H9 medium supplemented with 10% albumin-dextrose-catalase (ADC) (BD Sciences) and 0.05% Tween 80, in SCFM2 (Turner et al., 2015) devoid of mucin and DNA (medium referred to as SCFM in this study), or on Middlebrook 7H11 agar supplemented with 10% oleic acid-albumin-dextrose-catalase (OADC) (BD Sciences). To assess the impact of divalent cations on growth and biofilm formation, SCFM was prepared with CHELEX-100^®^-treated water (Bio-Rad) and FeSO_4_, ZnSO_4_, MnSO_4_, CuSO_4_, NiCl_2_, CoCl_2_, CaCl_2_, and MgCl_2_ were added at different concentrations.

### Biofilm Assay

*Mabs* NJH12 submerged biofilms were formed in 96-well (polystyrene, flat bottom) poly-D-lysine-coated plates in 200 μL of SCFM. Biofilm formation was monitored by colony-forming unit counting or by crystal violet staining as follows: Culture medium and planktonic cells were removed from 3 to 6-day-old biofilm plate and biofilms were washed gently with PBS prior to adding 100 μL of 0.05% crystal violet solution. After 30 min of incubation at room temperature, the wells were washed with PBS and crystal violet was extracted with 300 μL of 30% acetic acid for 30 min followed by reading the absorbance of the solution at 562 nm.

For fluorescence confocal imaging, *Mabs* NJH12 was transformed with pCHERRY3 (Addgene # 24659) expressing mCherry or pMSP12GFP (Chan et al., 2002) expressing GFP. mCherry and GFP-expressing *Mabs* NJH12 biofilms were grown for 3–6 days on poly-D-lysine-coated μ-Dish ^35*mm*,*low*^ (ibidi) at 37°C in a 5% CO_2_ incubator. Biofilms were stained using Nile Red (Invitrogen) for staining lipids, TOTO^TM^-1 iodide (Invitrogen) for nucleic acids, FilmTracer^TM^ SYPRO^®^ Ruby (Invitrogen) for proteins, and Texas Red^TM^ hydrazide (Invitrogen) for polysaccharides. Glass slides were visualized using a ZEISS LSM 510M ETA confocal microscope equipped with a 63x/1.40 plan-Apochromat objective. At least two independent experiments were performed, and images from one representative experiment are shown.

### Treatment of Established Biofilms With Enzymes

Enzyme treatments were performed on 4-day old biofilms. Planktonic cells were removed and biofilms were washed once with PBS prior to adding 150 μL of enzyme, or the corresponding buffer without enzyme as control. After 20 h of incubation at 37°C, planktonic cells were removed and the biofilm washed once with 150 μL PBS prior to quantification by crystal violet staining as described above. The following enzymes were used: TURBO^®^ DNase (Thermo Fisher Scientific) in the commercial buffer provided, Cellulase from *Trichoderma sp.* (Sigma) in 50 mM citrate buffer (pH 5.0), α-amylase from *Bacillus licheniformis* (Sigma) in PBS (pH 7.4), α-mannosidase from *Canavalia ensiformis* (Sigma) in 10 mM ammonium acetate buffer (pH 7.0), Proteinase K (GoldBio) in 10 mM Tris buffer (pH 8.0), Trypsin from bovine pancreas (Sigma) in PBS (pH 7.4), Lipase from *Candida rugosa* (Sigma) in PBS (pH 7.4), Lysozyme (Sigma) in PBS (pH 7.4) and phospholipases A1 (from *Aspergillus oryzae*; Sigma) and A2 (from *Apis mellifera*; Sigma) in 50 mM Tris–HCl (pH 7.5), 10 mM CaCl_2_ and 2% DMSO buffer.

### Minimum Inhibitory Concentration Determinations

The Minimum Inhibitory Concentrations (MICs) of a variety of antibiotics against *Mabs* NJH12 either grown planktonically or as biofilms were determined in SCFM, in a total volume of 200 μL in 96-well microtiter plates. For planktonically grown cells, *Mabs* NJH12 grown to early log phase were diluted to a final concentration of 10^6^ CFU mL^–1^ and incubated in the presence of serial dilutions of the drugs for 5 days at 37°C. MICs were determined using the resazurin blue test and read after 24 h (Martin et al., 2003). For biofilm-grown *Mabs* NJH12, serial dilutions of the antibiotics were added to 4-day old biofilms for 24 h and MICs were determined as described above. Rapid colorimetric methods based on Alamar blue, resazurin blue and water-soluble tetrazolium salts reflect metabolic activity and are extensively used to assay compound efficacy against replicating and non-replicating bacteria, including the viability of bacteria residing within biofilms (Marshall et al., 1995; Nett et al., 2011).

### Analysis of Extracellular Matrix Lipids, Sugars, Proteins, and Extracellular DNA

*Mabs* NJH12 triplicate cultures (50 mL each) were grown either planktonically under agitation (160 rpm) in SCFM for 2–5 days at 37°C, or as biofilms in the same medium in standing 175 cm^2^ poly-D-Lysine-coated tissue culture flasks at 37°C for 5 days. To provide a better reflection of the conditions encountered by the bacteria in the lung and thus ensure nutrients did not become limiting, the medium of biofilm and planktonic cultures was replaced with fresh medium on days 2, 3, and 4. Upon collection, bacterial pellets from planktonic and biofilm cultures were washed twice with sterile water, resuspended in sterile water with glass beads (4 mm in diameter) and vortexed for 1 min. The supernatants resulting from the centrifugation of these suspensions at 6,000 × *g* for 30 min yielded the ECM fractions which were subsequently freeze-dried and biochemically defined (see below). The pellet, corresponding to bacteria stripped of their ECM, was similarly freeze-dried and weighed.

For analysis of protein extracts, ECM were either submitted to SDS-PAGE analysis and Coomassie blue staining, or delipidated using a biphasic organic extraction with methyl tert-butyl ether:methanol:water [6:3:1, by vol.] followed by digestion using ProteaseMAX^TM^ Surfactant (Promega) in combination with trypsin. The digested samples were analyzed using a reverse phase HPLC coupled to a nanospray ionization source on an Orbitrap Velos mass spectrometer at Colorado State University’s Analytical Resources Core. The Mascot search engine was used to interpret the acquired MS/MS spectra by searching against Uniprot_*Mycobacterium*_*abscessus*_rev_021220 database, and the Scaffold proteomics software was applied to further validate the peptide identification and data interpretation. Peptide matches over 95% were considered as identified peptides. Proteins identified by more than two peptides were considered for further analysis. Two independent biofilm (day 5) and planktonic cultures (day 2 and day 5) were used for proteomics analyses. The experiment was done twice on independent cultures and representative results are shown.

Extracellular DNA (eDNA) in the ECM was quantified using the Qubit fluorometric quantification (Thermo Fisher Scientific).

Lipids were extracted from lyophilized ECM with CHCl_3_/H_2_O (1:1, v/v) and the lipids recovered from the chloroform phase subsequently analyzed by thin-layer chromatography (TLC) on aluminum-backed silica gel 60-precoated plates F254 (E. Merck), or by LC-MS in positive and negative ion modes following the method described by Sartain et al. (2011) on an Agilent 1260 Infinity chromatograph equipped with a 2.1 mm × 150 mm (3.5 μm particle size) XBridge reverse phase C18 column (Waters) coupled to an Agilent 6224 time-of-flight (TOF) mass spectrometer. Data analysis was carried out using the Agilent MassHunter software.

To determine the monosaccharide composition of the ECM, the water phase retained after the extraction of lipids with chloroform was freeze-dried and alditol acetate derivatives of monosaccharides were prepared following an earlier procedure (Kaur et al., 2007). Alditol acetates were analyzed by gas chromatography-mass spectrometry (GC/MS) on a Thermo Scientific TRACE 1310 Gas Chromatograph paired with a Thermo Scientific TSQ 8000 Evo Triple Quadrupole GC-MS/MS. Samples were run on a Zebron ZB-5HT Inferno 30 m × 0.25 mm × 0.25 μm capillary column (Phenomenex) at an initial temperature of 100°C. The temperature was increased to 150°C at a ramp rate of 20°C min^–1^, then to 240°C at a ramp rate of 5°C min^–1^ and was held at this temperature for 3 min to be finally increased to 300°C at a rate of 30°C min^–1^ and held at the final temperature for 5 min. Collected data were analyzed using the Thermo Scientific Chromeleon Chromatography Data System software.

### RNA Extraction, Reverse Transcription, and RT-qPCR

Two independent biofilm (day 2 and day 5) and planktonic (day 1 and day 2) cultures were used for transcriptomics analyses. RNA extraction with the Direct-zol^TM^ RNA Miniprep kit (Zymo Research), reverse transcription reactions using the Superscript IV First-Strand Synthesis System (Thermo Fisher Scientific) and RT-qPCRs using the SsoAdvanced^TM^ Universal SYBR^®^ Green Supermix (Bio-Rad) were conducted per the manufacturers’ protocols and analyzed on a CFX96 real-time PCR machine (Bio-Rad). PCR conditions: 98°C (30 s; enzyme activation), followed by 40 cycles of 98°C (10 s; denaturation) and 60°C (30 s; annealing/extension). Mock reactions (no reverse transcription) were done on each RNA sample to rule out DNA contamination. The target cDNA was normalized internally to the *sigA* cDNA levels in the same sample. The following primers were used: MAB_0663_Fw (5′-GAACTGGCGGCATTCATGTG-3′), MAB_ 0663_Rv (5′-CTGGATGTCGATCGTGCTGA-3′); MAB_2122 (mbtE)_Fw (5′-TGGCAGAGCGAAAGTCCAAT-3′), MAB_2122 (mbtE)_Rv (5′-AAGCTATCGTCCGGGTTC AC-3′); MAB_3354_Fw (5′-CCGACCTCGAGCTCCTACAC-3′), MAB_3354_Rv (5′-GGATGTAGTCGTGCGGGTTCC-3′); MAB_2142 (nuoI)_Fw (5′-ATGCCTGATCTTCTGCGACG-3′), MAB_2142 (nuoI)_Rv (5′-AGCCGCTTCTTGAACATGGA- 3′); MAB_2233c (eccB3)_Fw (5′-CAGTGCCCTCAATCTCG GAG-3′), MAB_2233c (eccB3)_Rv (5′-TTGAGCAGACGAGT GGTGAC-3′); MAB_3731c (groEL1)_Fw (5′-GACAAGGGCT ATCTGTCGCA-3′); MAB_3731c_Rv (5′-CTGATCTTGTCGCG ATGCAG-3′); and *sigA*_fwd (5′-CGTTCCTGGACCTGATTC AG-3′), *sigA_rev* (5′-GTACG TCGAG AACTT GTAACCC-3′).

### RNA-Seq Library Preparation

RNA was quantified using a Qubit RNA spectrophotometer (Thermo Fisher) and sample quality was assessed using an Agilent High Sensitive RNA Screentape on an Agilent Tapestation, according to the manufacturer’s recommendations. All RNA had an RNA Integrity Number (RIN) of greater than 6, indicating sufficient RNA quality for sequencing. Ribosomal depletion was performed using an adapted protocol from Huang et al. (2020) ssDNA oligo probes were designed to cover *Mabs* 16S and 23S rRNA using the RNaseH_depletion scripts^1^ developed by Huang et al. (2020). The ssDNA oligo probe sequences are available in Supplementary Table 1. The oligo probe library was chemically synthesized (Integrated DNA Technologies Inc.), resuspended in a plate format (100 μM), and equimolarly pooled to generate the oligo probe mix used in this study. Ribosomal RNA was depleted from 0.5 μg of total RNA using a 5X probe ratio and 3 μL Hybridase Thermostable RNase H (Lucigen) as recommended by Huang et al. (2020). Probes were then removed by DNase treatment (Thermo Fisher Scientific) followed by a 2X bead clean-up (AMPure RNA, Beckman Counter). Depleted RNA was resuspended in the fragmentation buffer provided with the KAPA RNA Hyperprep kit (Roche). Fragmentation was performed for 6 min at 85°C followed by the 1st and 2nd strand synthesis. Ligation was performed with 1.5 μM of KAPA Dual-Indexed Adapter (Roche). After the final amplification step (5 cycles), libraries were quantified using Qubit dsDNA BR Assay Kit (Thermo Fisher Scientific, Waltham, MA, United States), and the fragment size was assessed on an Agilent Tapestation using the D1000 Screen tape. Libraries were multiplexed on one sequencing run at equimolar concentrations. Libraries were sequenced using single-end or pair-end reads on an Illumina NextSeq instrument using the mid-output 75 cycles.

### RNA-Seq Data Analysis

RNA-seq reads were trimmed for quality score greater than 20 and length greater than 50 using Skewer (version 0.2.2) automatically detecting adapters (Jiang et al., 2014). Reads were mapped to the *Mabs* subsp. *abscessus* ATCC 19977 genome (NC_010397.1) using Bowtie 2 (version 2.3.5) end-to-end alignment with default parameters (Langmead and Salzberg, 2012). Count tables were constructed from sorted BAM files using HTSeq-count (version 0.11.1) (Anders et al., 2015) set to non-stranded, intersection_nonempty using the gff3 file for NC_010397.1 and counting reads on gene_id. Gene expression and differential expression analysis was completed in R (version 3.6.0) using DESeq2 (version 1.26.0) (Love et al., 2014). Genes were identified as differentially expressed if they had a fold change ≥2.0 and Benjamini-Hochberg multiple testing correction adjusted *p*-value of 0.05 or less.

### Functional Enrichment Analysis

We mapped the significantly differentially expressed genes in the comparison B2 vs. P1 and B5 vs. P2 to genome-scale metabolic network construction of *M. tuberculosis* H37Rv by identifying orthologs using protein-to-protein sequence comparison using the OMA and the BLOSUM62 scoring matrix (Pearson, 2013). Then, we identified metabolic pathways that were significantly enriched in the *Mabs* biofilm stages [Benjamini Hochberg corrected for false discovery rate (FDR) *p*-value < 0.05 (Benjamini and Hochberg, 2000) and Fold-enrichment > 2] using the KEGG and Gene Ontology (GO) databases in the DAVID’s functional annotation tool (Huang et al., 2009).

### Data Availability

The sequencing data described in this publication have been submitted to the NCBI gene expression omnibus (GEO) under BioProject # PRJNA648126.

## Results

### Formation of *Mycobacterium abscessus* Complex Biofilms in Synthetic Cystic Fibrosis Medium

**FIGURE 1:**
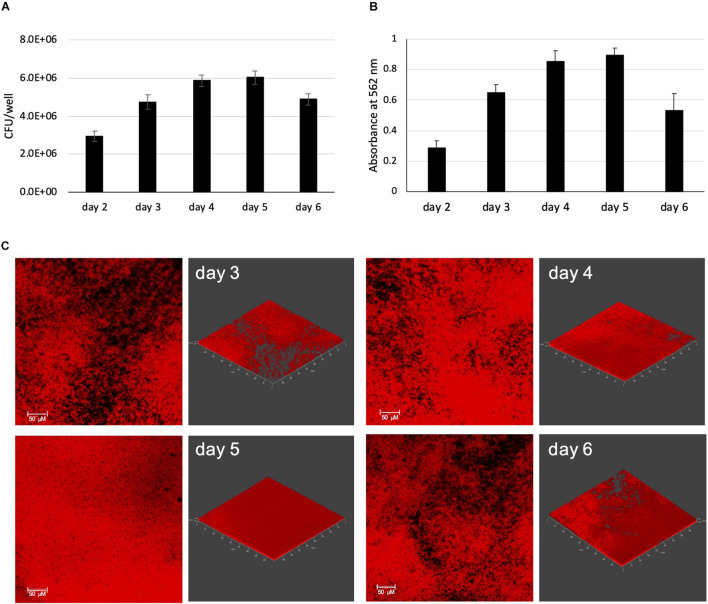
MABSC biofilm formation in synthetic CF medium. *Mabs* NJH12 biofilm formation as monitored by CFU counting **(A)** and Crystal violet staining **(B)**. Shown are averages ± standard deviations of triplicate wells containing 200 μL of SCFM. **(C)** Development of mCherry-expressing *Mabs* NJH12 biofilms as monitored by fluorescence confocal imaging. 2D (left panels) and 3D (right panels; X × Y × Z = 497 μm × 497 μm × 12 μm) views of the biofilms are shown. The density of biofilms increases over time until they reach maturation on day 5 and start detaching from the substratum on day 6.

To establish an *in vitro* model of biofilm formation that mimics as closely as possible the environment encountered by MABSC in the airway and lung of persons with CF, we used a modified synthetic CF medium (herein referred to as SCFM) based on the composition of expectorated sputum (Turner et al., 2015) to grow MABSC biofilms in 96-well poly-D-lysine-coated plates. We recently showed this medium to closely mimic the metabolic adaptation undergone by MABSC in actual CF sputum (Wiersma et al., 2020). MABSC grows as submerged biofilms firmly attached to the bottom of the wells in this model allowing for reproducible quantification of biofilm formation by CFU counting (Figure 1A) and crystal violet staining (Figure 1B) over time. Consistent with an earlier report (Clary et al., 2018), biofilm formation occurred rapidly with measurable crystal violet staining and CFU counting 2 days post-inoculation. In the modified synthetic CF medium used herein, mucin was omitted to allow quantification of biomass by crystal violet staining, and so was eDNA to allow DNA originating from the bacilli to be detected in subsequent ECM analyses. Representative confocal images of biofilms from *Mabs* NJH12, a clinical isolate from a patient with CF, are shown in Figure 1C. As expected, biofilm growth in this model results in bacteria displaying higher tolerance to a panel of clinically used antibiotics compared to planktonically grown cells, including amikacin, azithromycin, ciprofloxacin, clofazimine, cefoxitin, and imipenem (Table 1).

**TABLE 1:** Minimal inhibitory concentrations (MICs) of a variety of antibiotics against planktonically- and biofilm-grown *Mabs* NJH12 in synthetic CF medium.

Antibiotic	AMI	AZI	CIP	CFZ	CEF	IMI
Planktonic	16	2	8	0.5	16	8
Biofilm	>256	>256	>256	>256	>256	>256

*Mabs NJH12 was grown in SCFM under planktonic and biofilm conditions. MIC determinations (in μg/mL) were performed using the resazurin blue test and are representative of two independent experiments.*

### Confocal Microscopy Imaging of *Mycobacterium abscessus* Complex Biofilms Stained With Specific Dyes

The hallmark of biofilms is the production of an ECM that holds the community of bacteria together and contributes to drug tolerance and phenotypic heterogeneity. Important constituents of the ECM identified in a variety of *Mycobacterium* species include proteins, eDNA, mycolic acids, glycopeptidolipids (GPLs), polysaccharides and other more or less defined cell envelope constituents (Richards and Ojha, 2014; Xiang et al., 2014; Rose et al., 2015; Rose and Bermudez, 2016; Trivedi et al., 2016; Chakraborty and Kumar, 2019; Chakraborty et al., 2021).

**FIGURE 2:**
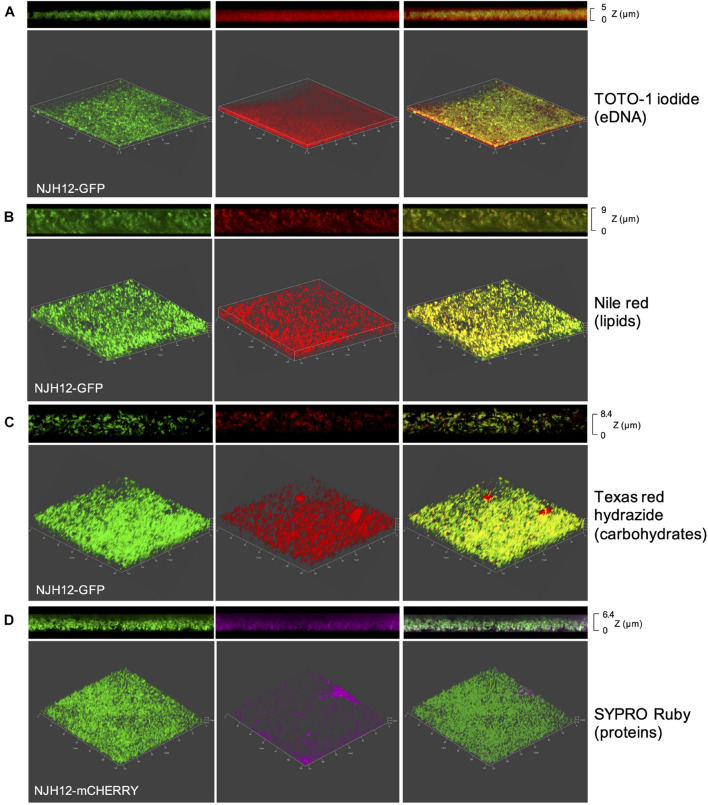
Staining of MABSC biofilms with protein, lipid, polysaccharide, and DNA-specific dyes. mCherry- or GFP-expressing *Mabs* NJH12 biofilms were stained with TOTO-1 iodide for staining eDNA **(A)**, Nile red for staining intra- and extra-cellular lipids **(B)**, Texas Red hydrazide for staining extracellular polysaccharides **(C)** and SYPRO Ruby for staining extracellular proteins **(D)**. Shown are three-dimensional (bottom; X × Y × Z = 128 μm × 128 μm × 5–9 μm) and side views (top) of the stained biofilms as analyzed by fluorescence confocal imaging. Left panel: mCherry- or GFP-expressing *Mabs* NJH12. Middle panel: constituent-specific staining of the biofilm; Right panel: overlay of the previous images.

The content of the ECM of SCFM-grown MABSC biofilms was first investigated by staining 5-day old biofilms formed by *Mabs* NJH12 expressing GFP or mCherry with different dyes specific for eDNA, proteins, lipids or carbohydrates, followed by fluorescence confocal imaging. Staining with TOTO-1 iodide which does not stain intracellular DNA clearly revealed the presence of abundant quantities of eDNA in the ECM of SCFM-grown *Mabs* NJH12 biofilms (Figure 2A). Staining with Nile red which stains both intracellular and extracellular lipids clearly revealed the presence of lipids co-localizing with the bacilli as well as within the ECM of bacterial clusters (Figure 2B). A similar staining pattern was obtained with Texas Red hydrazide which intensely labeled both the bacilli and areas of the ECM indicative of the presence of polysaccharides at the bacilli’s surface and within the ECM (Figure 2C). Finally, staining with the protein-specific dye FilmTracer^TM^ SYPRO^®^ Ruby revealed extracellular proteins as abundant components of the ECM although their distribution differed from that observed with eDNA, lipids and polysaccharides in that labeling was most intense within deeper layers of the biofilms, closer to the substratum, and showed no clear evidence of co-localization with the bacilli (Figure 2D). A control run without biofilm in the well did not suggest any bias in the results caused by the staining of poly-D-Lysine by SYPRO^®^ Ruby (data not shown).

### Biochemical Definition of the Extracellular Matrix of *Mycobacterium abscessus* Complex Biofilms

To biochemically define the composition of the ECM from SCFM-grown *Mabs* NJH12 biofilms, triplicate cultures of *Mabs* NJH12 were grown in poly-D-lysine-coated tissue culture flasks for 5 days at 37°C, replacing the supernatant with fresh SCFM on days 2, 3, and 4 so that nutrients did not become limiting. Parallel triplicate *Mabs* NJH12 cultures were planktonically grown in SCFM under agitation at 37°C for 2–5 days until they reached late exponential phase and stationary phase, respectively, with medium replacement on days 2, 3, and 4 (Supplementary Figure 1). The ECM fraction from biofilm and planktonically grown cells was isolated by mechanical disruption with glass beads, a method known not to affect the integrity of the cells (Ortalo-Magné et al., 1995; Marsollier et al., 2007), prior to freeze-drying and further analyzing carbohydrates, lipids, DNA and proteins.

**FIGURE 3:**
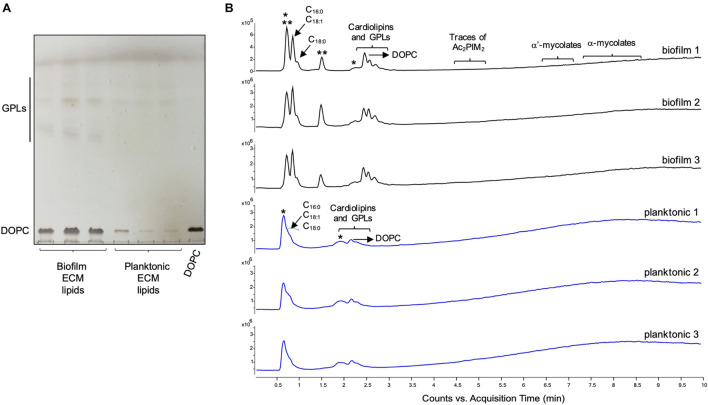
Lipid composition of the ECM from planktonically grown and biofilm-grown *Mabs* NJH12. **(A)** Thin-layer chromatography analysis of ECM lipid fractions prepared from *Mabs* NJH12 extracellular matrices. 1/30^th^ of the total lipids prepared from 150 mL cultures of day 2 planktonically grown cells (corresponding to 1.95 ± 0.02 mg of dry bacilli devoid of ECM) and 150 mL cultures of day 5 biofilm-grown cells (corresponding to 1.11 ± 0.03 mg of dry bacilli devoid of ECM) was loaded and the plate was developed in chloroform:methanol:water (20:4:0.5 by vol.). Lipids were revealed by spraying with cupric sulfate (10% CuSO_4_ in 8% phosphoric acid solution) and charring. The lipid fractions from triplicate cultures are shown. Pure DOPC control (250 ng) was loaded in the last lane. **(B)** Negative mode LC/MS analysis of the lipid fractions prepared from triplicate ECM preparations of biofilm-grown (black lines) (day 5) and planktonically grown (blue lines) (day 2) *Mabs* NJH12. Total ion chromatograms are shown. See Table 3 for the individual molecular species identified, their corresponding mass accuracy (ppm) values, and relative abundance in the planktonic and biofilm ECM samples. Single and double asterisks denote unknown, co-eluting, compounds. Peaks marked by double asterisks (**) were specifically seen in the ECMs prepared from biofilm cultures. Ac_2_PIM_2_, tetraacylated phosphatidylinositol dimannosides; DOPC, dioleoyl phosphatidylcholine (from SCFM); GPL, glycopeptidolipids.

The ECM recovered from 5-day-old biofilm-grown *Mabs* NJH12 represented as much as 30% of the total weight of the biofilm which was ∼ 4 times the amount of ECM recovered from planktonically grown cells (7.4%) (average of three biological replicates for each culture condition). Compositional analysis indicated that while proteins represented the bulk of the ECM material prepared from planktonically grown cells, lipids dominated the composition of the ECM of *Mabs* NJH12 biofilms, followed by proteins, polysaccharides and eDNA (Table 2). Looking at sugar:protein:lipid:eDNA ratios, the most striking difference between the two culture conditions was a 10-fold increase in the eDNA and lipid contents of the ECM of biofilm-grown cells compared to planktonically grown cells (Table 2). An analysis of ECM lipids by TLC, however, indicated that DOPC was the main lipid constituent. Thus, MABSC used the phospholipid available in SCFM to incorporate into its biofilm matrix more than it actively produced and secreted endogenous lipids to promote biofilm development (Figures 3A,B). In contrast, the dramatic increase in the abundance of eDNA found in the ECM of biofilm-grown bacilli relative to other constituents, clearly pointed to the active release of DNA by the bacteria as the biofilm develops.

**TABLE 2:** Composition of the ECM of planktonically- and biofilm-grown *Mabs* NJH12 in synthetic CF medium.

	ECM planktonic cells (μg/mg dry cells)	ECM biofilms (μg/mg dry cells)
Sugars	3.08 ± 0.27	5.85 ± 3.48
Proteins	13.07 ± 1.60	28.07 ± 2.08
Lipids	1.88 ± 0.45	36.96 ± 6.24
eDNA	0.11 ± 0.005	2.04 ± 0.21
[sugar:protein:lipid:eDNA] ratio	1:4.24:0.61:0.035	1:4.80:6.32:0.35

*The ECM of 2 days-old planktonically grown cells and 5 days-old biofilm-grown Mabs NJH12 cells was collected and processed for DNA, lipid, protein, and sugar quantitative and qualitative analysis as described under section “Materials and Methods.” Bacterial cells recovered after their ECM was removed were freeze-dried and weighed. The amounts of ECM-derived DNA, lipids, proteins, and sugars provided below are expressed relative to the dry weight of bacilli stripped of their ECM. Shown are averages ± SD of triplicate cultures.*

To gain further insights into the nature of the MABSC lipids found in the ECM of planktonically- and biofilm-grown cells, lipid fractions were submitted to LC/MS analysis. The results not only revealed shared constituents between the two types of ECM, but also highlighted some lipids whose abundance markedly increased under biofilm growth conditions (Figure 3B and Table 3). Predominant shared lipids included DOPC, various forms of di- and tri-glycosylated GPLs (Wiersma et al., 2020), free fatty acids (palmitic, palmitoleic, and oleic acids) and cardiolipin. Lipids found exclusively or in greater abundance in the ECM of biofilm-grown cells included some forms of GPLs, free mycolic acids and tetraacylated forms of phosphatidylinositol dimannosides (Ac_2_PIM_2_) as well as some constituents (marked by double asterisks in Figure 3B) whose *m/z* did not yield any identifiable compound in the lipidomics database used herein (Sartain et al., 2011). In contrast to the situation in *M. smegmatis*, *M. tuberculosis*, *M. chelonae*, and MABSC biofilms grown at the air-liquid interface in minimal laboratory media (Ojha et al., 2005, 2008; Ojha and Hatfull, 2007; Vega-Dominguez et al., 2020; Dokic et al., 2021), free mycolates were relatively minor constituents of the ECM of SCFM-grown MABSC biofilms (Figure 3B) and the mycolate species identified did not differ between biofilm- and planktonically grown cells. No trehalose dimycolates were detected under any of the two growth conditions.

**TABLE 3:** Lipid composition of the ECM of planktonically- and biofilm-grown *Mabs* NJH12.

Structures	Theoretical mass (*m/z*)	Biofilm	Planktonic	Charge	Observed mass *(m/z)*	Mass accuracy (ppm)
**DOPC** 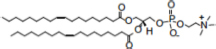	844.6073	+	+	[M+Hac-H]^–^	844.6095	2.58

**Free fatty acids**	253.2173 (C_16:1_)	+	+	[M-H]^–^	253.2175	0.67
Palmitoleic acid (C_16:1_), Palmitic acid (C_16:0_)	255.2329 (C_16:0_)	+	+	[M-H]^–^	255.2322	–2.74
Oleic acid (C_18:1_), Stearic acid (C_18:0_)	281.2486 (C_18:1_)	+	+	[M-H]^–^	281.2471	–5.33
	283.2642(C_18:0_)	+	+	[M-H]^–^	283.2657	5.30

**Free mycolic acids** 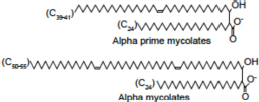	913.9321	+	nd	[M-H]^–^	913.9374	5.76
	927.9478	t	–	[M-H]^–^	927.9495	1.83
	941.9634	+	+	[M-H]^–^	941.9632	–0.27
	1080.1040	+	–	[M-H]^–^	1080.1071	2.86
	1108.1354	+	t	[M-H]^–^	1108.1313	–3.65
	1122.1511	+	t	[M-H]^–^	1122.1491	–1.76
	1136.1668	+	t	[M-H]^–^	1136.1641	–2.34
	1150.1825	+	t	[M-H]^–^	1150.1812	–1.08
	1164.1982	t	+	[M-H]^–^	1164.1892	–7.68

**Cardiolipins** 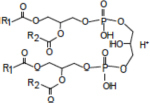	1265.8543 (2R_1_ + 2R_2_ = 58:2)	+	+	[M-H]^+^	1265.8517	–2.01
	1279.8699 (2R_1_ + 2R_2_ = 59:2)	+	+	[M-H]^+^	1279.8629	–5.46
	1293.8856 (2R_1_ + 2R_2_ = 60:2)	+	+	[M-H]^+^	1293.8959	7.99
	1307.9012 (2R_1_ + 2R_2_ = 61:2)	+	+	[M-H]^+^	1307.8983	–2.22
	1321.9169 (2R_1_ + 2R_2_ = 62:2)	+	+	[M-H]^+^	1321.9147	–1.62
	1335.9325 (2R_1_ + 2R_2_ = 63:2)	+	+	[M-H]^+^	1335.9302	–1.74
	1349.9482 (2R_1_ + 2R_2_ = 64:2)	+	+	[M-H]^+^	1349.9412	–5.16
	1363.9638 (2R_1_ + 2R_2_ = 65:2)	+	+	[M-H]^+^	1363.9522	–8.48
	1377.9795 (2R_1_ + 2R_2_ = 66:2)	+	+	[M-H]^+^	1377.9729	–4.77

**Glycopeptidolipids (GPLs)** 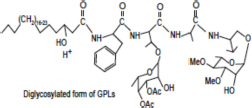	1207.7939	+	+	[M-H]^+^	1207.7970	2.58
	1221.8096	+	+	[M-H]^+^	1221.8110	1.19
	1235.8253	+	+	[M-H]^+^	1235.8347	7.66
	1249.8410	+	+	[M-H]^+^	1249.8458	3.86
	1263.8567	+	+	[M-H]^+^	1263.8620	4.18
	1277.8724	+	+	[M-H]^+^	1277.8756	2.51
	1291.8881	+	+	[M-H]^+^	1291.8879	–0.10
	1305.9038	+	–	[M-H]^+^	1305.9042	0.29

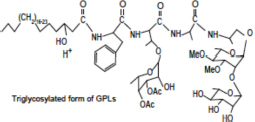	1353.8518	+	+	[M-H]^+^	1353.8574	4.16
	1367.8677	+	+	[M-H]^+^	1367.8632	–3.28
	1381.8834	+	+	[M-H]^+^	1381.8844	0.74
	1395.8991	+	+	[M-H]^+^	1395.9031	2.89
	1409.9148	+	+	[M-H]^+^	1409.9119	2.04
	1423.9305	+	+	[M-H]^+^	1423.9345	2.84

**Ac_2_PIM_2_** 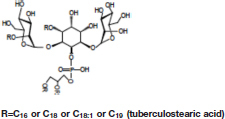	1622.0835	t	–	[M-H]^–^	1622.0758	–2.75
	1636.0992	t	–	[M-H]^–^	1636.1103	6.81
	1648.0992	t	–	[M-H]^–^	1648.0992	0.01
	1650.1148	t	–	[M-H]^–^	1650.1112	–2.19
	1662.1148	t	–	[M-H]^–^	1662.1222	4.45

*LC-MS analyses were run in both positive and negative modes and the m/z values of the main compounds identified are shown. Plus and minuses refer to the presence or absence of detectable amounts of lipid constituents. t denotes ions present at trace levels (counts < 100).*

**FIGURE 4:**
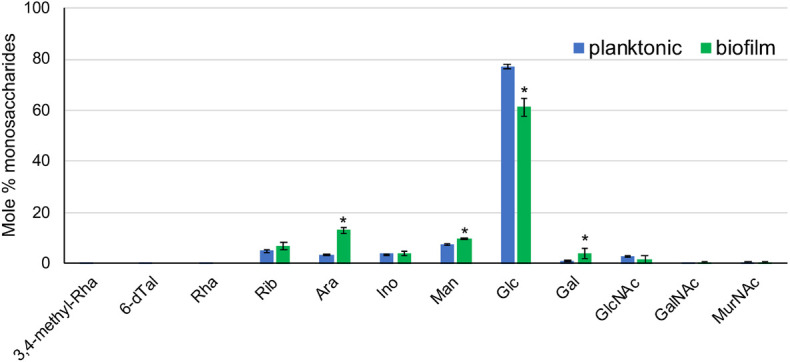
Sugar composition of the ECM from planktonically grown and biofilm-grown *Mabs* NJH12. Monosaccharidic composition of *Mabs* NJH12 planktonic and biofilm extracellular matrices. The reported values are averages ± standard deviations of three biological replicates and represent relative mole percentages. Asterisks denote statistically significant differences between the two culture conditions pursuant to the Student’s *t*-test (*p* < 0.05).

Sugar analysis identified glucose as the main monosaccharide present in the ECM of *Mabs* NJH12 grown under both culture conditions followed by mannose, arabinose, ribose, galactose, inositol, *N*-acetyl-glucosamine, and *N*-acetyl muramic acid (Figure 4). We note that both glucose and *N*-acetyl-glucosamine are present in SCFM and could therefore, at least in part, be coming from residual traces of the culture medium in our ECM preparations. Alternatively, cellulose was recently reported to be a structural component of NTM biofilms grown in laboratory medium under thiol reductive stress (Chakraborty et al., 2021) and, along with capsular α-D-glucan (Ortalo-Magné et al., 1995), may represent a source of ECM glucose. The fact that 3 to 4 times more arabinose and galactose were found in the ECM of biofilm-grown cells compared to that of planktonically grown cells is either suggestive of the release of arabinogalactan from lysed cells within the biofilm or of the presence of as yet uncharacterized arabinan- and galactan-based exopolysaccharide(s) in the biofilm matrix of *Mabs* as recently proposed in *M. smegmatis* and *M. tuberculosis* (Bharti et al., 2020).

Coomassie blue staining of an SDS-PAGE gel of proteins prepared from total bacilli and ECMs from planktonically- and biofilm-grown *Mabs* NJH12 revealed significantly different profiles (Supplementary Figure 2). ECM proteins from both culture conditions were submitted to proteomics analysis. Twenty-seven proteins were uniquely found in the ECM from *Mabs* NJH12 biofilms and 75 proteins were found in significantly greater abundance in this preparation compared to the ECM prepared from planktonically grown cells (*p* < 0.05) (Supplementary Table 2). Overall, unique or enriched proteins of the biofilm ECM were overwhelmingly comprised of proteins involved in central carbon metabolism, respiration (ATP synthase, cytochrome c oxidase, ferredoxin; type II NADH dehydrogenase), amino acid and lipid metabolism, and heat shock responses (Clp proteases; chaperones: GroEL, GroES, DnaK, ClpB, 18 kDa antigen) (Supplementary Table 2). Interestingly, a catalase (MAB_0351) involved in response to oxidative stress, and a lipase (MAB_2814) potentially involved in the utilization of DOPC from SCFM were found in significantly greater abundance in the ECM of biofilm-grown cells. Also worth mentioning among the proteins uniquely found in biofilm ECM are (i) two DNA-binding proteins (MAB_0019; MAB_2883c) which may play a role in stabilizing the matrix upon DNA release by the bacilli; (ii) five proteins predicted to belong to the hypoxia-induced DosRS regulon including DosR itself [MAB_1041, MAB_2489, MAB_3354; MAB_3891c (DosR) and MAB_3902c] (Park et al., 2003; Gerasimova et al., 2011), and (iii) an enzyme involved in arabinan synthesis (MAB_1147c) which may participate in the formation of ECM polysaccharides. We finally note that more than 20 of the proteins found to be enriched in the ECM of biofilm-forming *Mabs* NJH12, including 8 out of 27 proteins uniquely found in the biofilm ECM, were also found by transcriptional profiling to be expressed at a significantly higher level in biofilms than in planktonically grown bacilli (see further in the text) (see proteins labeled with an asterisk in Supplementary Table 2).

### Dispersal of Established *Mycobacterium abscessus* Complex Biofilms by DNase, Glycosidases, and Phospholipases

**FIGURE 5:**
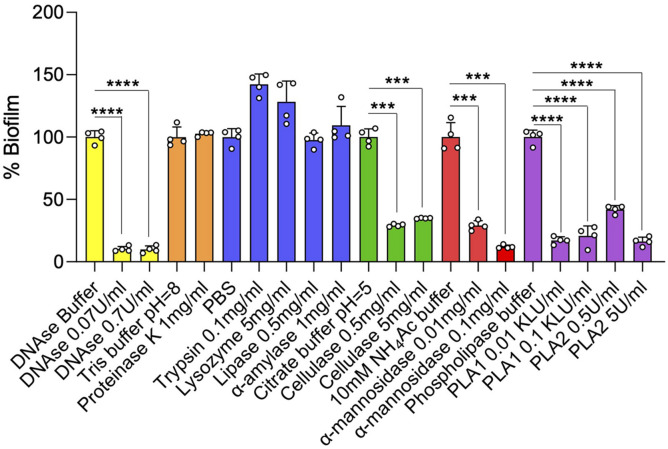
Effect of enzyme treatments on MABSC biofilm dispersal. Mature 4-day-old biofilms were treated with DNase, lipase, phospholipases A1 (PLA1) and A2 (PLA2), cellulase, α-amylase, α-mannosidase, lysozyme, proteinase K, or trypsin for 20 h at 37°C. The biofilms remaining after treatment were washed and quantified by crystal violet staining. The values are expressed as percentages of the values obtained for the controls (treatment with PBS or other enzyme buffers alone). Biofilm assays run in the same buffer are colored similarly. Quantification was performed on four wells per treatment. Two independent experiments were performed with similar results. Asterisks denote statistically significant differences between enzyme treatment and control groups (enzyme buffer without enzyme) pursuant to the Student’s *t*-test (***p < 0.0005, ****p < 0.00005).

Consistent with the finding of DNA in the ECM of SCFM-grown *Mabs* NJH12 biofilms, but in contrast to recent conclusions drawn from the analysis of the ECM of MABSC biofilms grown in minimal Sauton’s medium (Chakraborty et al., 2021), the addition of DNase on established 4-day-old NJH12 biofilms caused a significant dispersal of the biofilm (Figure 5). A similar effect was observed with α-mannosidase and cellulase indicating that glucose- and mannose-based (lipo)polysaccharides (i.e., possibly lipomannan and/or lipoarabinomannan, capsular mannan and/or arabinomannan, and cellulose) play an important role in the organization of MABSC biofilms. Likewise, phospholipase A1 and A2 treatment, which we confirmed to hydrolyze DOPC *in vitro* (data not shown), led to the dispersal of the biofilms in line with the abundant quantities of phospholipids (DOPC and cardiolipin) found in the ECM of biofilm-grown cells (Figure 3 and Table 3). A triglyceride-specific lipase, in contrast, had no effect on pre-established biofilms (Figure 5) consistent with the fact that no detectable amounts of triglycerides were found in the ECM. The lack of effect of proteases (proteinase K and trypsin) on biofilm dispersal despite the abundance of proteins in the ECM of biofilm-grown cells (Table 2) indicates that proteins do not significantly contribute to holding the clusters of bacilli together although one cannot exclude that they play a role at a different stage of the development of the biofilm (e.g., attachment or maturation). Finally, α-amylase and lysozyme also had no significant effect on pre-established biofilms (Figure 5). Again, the ineffectiveness of proteases on established MABSC biofilms contrasts with the potent dispersing activity of proteinase K on MABSC biofilms formed in Sauton’s medium (Chakraborty et al., 2021) and highlights the variations in composition undergone by MABSC biofilms depending on the environment in which they are grown.

Interestingly, omitting DOPC from the composition of SCFM, without significantly affecting growth (Supplementary Figure 1), appeared to facilitate biofilm formation by allowing earlier attachment to the substratum (Supplementary Figure 3) and the formation of what appeared to be (on pipetting) more firmly attached biofilm structures. We thus conclude that while impairing the initial attachment of MABSC biofilms to substratum, the host surfactant lipids that become incorporated in the ECM subsequently contribute to holding the clusters of bacilli together.

### Transcriptional Changes Associated With *Mycobacterium abscessus* Complex Biofilm Formation and Maturation

To analyze transcriptional changes associated with biofilm formation and maturation, *Mabs* NJH12 grown planktonically for 1 or 2 days [mid (P1) and late (P2) log phase, respectively; see Supplementary Figure 1] or as biofilms in our SCFM model for 2–5 days [early-stage surface attachment (B2) to mature (B5) biofilm, respectively; see Figure 1] were collected and their RNA subjected to RNA-sequencing. Two time points were studied for each culture conditions to analyze separately early- and late-stage biofilms and more specifically identify those differentially expressed genes involved in biofilm development and maturation from those whose expression was merely impacted by aging. To ensure nutrients did not become limiting in the biofilm cultures incubated for 5 days, the medium was replaced on days 2, 3, and 4 with fresh SCFM. Based on the growth curve of *Mabs* NJH12 in SCFM presented in Supplementary Figure 1, nutrients in planktonic cultures did not become limiting until after day 2.

**FIGURE 6:**
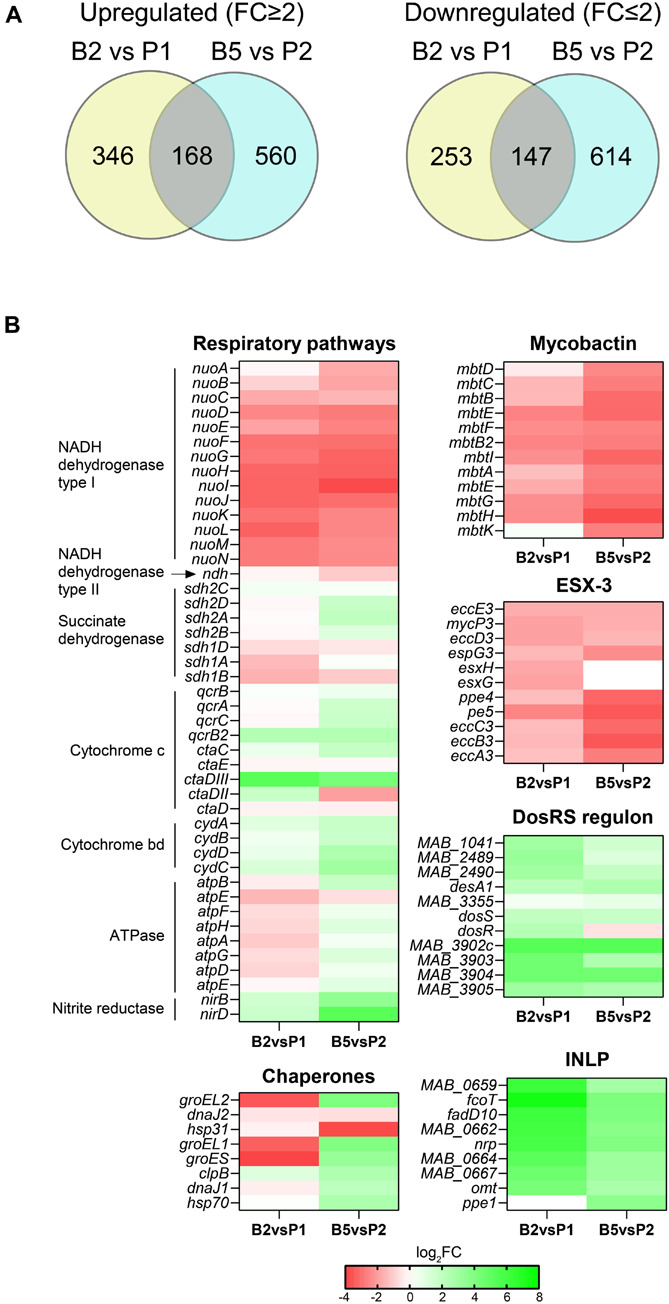
Transcriptional profile of MABSC residing in biofilms. **(A)** Venn diagrams showing the number of genes expressed at significantly higher or lower levels in *Mabs* NJH12 biofilms at day 2 vs. *Mabs* NJH12 biofilms planktonic cultures at day 1 (B2 vs. P1) or biofilms at day 5 vs. planktonic cultures at day 2 (B5 vs. P2). **(B)** Heatmap showing changes in the expression of genes involved in a few selected pathways found to be enriched in our analyses. INLP genes are involved in the biosynthesis of isonitrile lipopeptides. ESX-3 and mycobactin are involved in iron acquisition. The DosR regulon is involved in the response to hypoxia and oxidative stress. The results of our complete functional enrichment analysis are presented in Supplementary Figure 4.

Analysis of differentially expressed (DE) genes (fold change ≥2.0 and ≤−2.0 with a FDR adjusted *p*-value < 0.05) revealed 514 and 400 upregulated genes, and 760 and 761 downregulated genes when comparing B2 vs. P1 and B5 vs. P2, respectively. Of these genes, 168 were expressed at a higher level and 147 were expressed at a lower level under both conditions (Figure 6A and Supplementary Table 3). The metabolic pathways associated with the DE genes in B2 vs. P1 and B5 vs. P2 and those specific to biofilms in both comparisons were assessed using pathway enrichment in comparison to *M. tuberculosis* metabolism and the results are reported in Supplementary Figure 4.

Interestingly, genes predicted to belong to the hypoxia-responsive DosRS regulon of MABSC (Gerasimova et al., 2011; Miranda-CasoLuengo et al., 2016) were among the most significantly upregulated at both stages of biofilm formation (B2 vs. P1 and B5 vs. P2) (Figure 6B). This is reflective of the microaerophilic conditions to which biofilm-growing cells become exposed and is consistent with the concomitant differential expression of multiple other genes related to respiration and oxidative stress (Figure 6B and Supplementary Figure 4), including some encoding a pyruvate dehydrogenase and TCA cycle enzymes. This adaptation to biofilm growth in SCFM has precedence in the metabolic rewiring undergone by *Pseudomonas aeruginosa* in the CF airway (Rossi et al., 2021).

Other genes most significantly induced at one or both stages of biofilm formation (B2 vs. P1 and/or B5 vs. P2) included genes involved in isonitrile lipopeptide (INLP) biosynthesis (Figure 6B; Harris et al., 2017), carbonic anhydrase encoding genes (e.g., *MAB_0468*, *MAB_3211c*, and *MAB_2564*), lipase and esterase genes (e.g., *MAB_4134* and *MAB_2484*) and a possible Mce gene cluster of unknown function (*MAB_1693* through *MAB_1699*) that was induced 6.2–7.2 log_2_-fold. In *M. avium*, carbonic anhydrases have been shown to be induced upon exposure to bicarbonate and to positively influence eDNA export (Rose and Bermudez, 2016). Likewise, INLP were found to be important to the architecture development of *M. tuberculosis* biofilms suggestive of a generalized role of these secondary metabolites in the formation of mycobacterial biofilms (Richards et al., 2019), in addition to a possible role in metal transport and homeostasis in *M. marinum* (Harris et al., 2017), and virulence in tuberculous mycobacteria (Hotter et al., 2005; Dhar and McKinney, 2010; Bhatt et al., 2018). Unfortunately, the structure of INLP has only been partially characterized in *M. marinum* and *M. tuberculosis* thus far (Harris et al., 2017; Richards et al., 2019) and we were not able to detect any of the forms reported in the literature in the ECM of *Mabs* NJH12 by LC/MS, either due to their low production levels or to the fact that MABSC produces structurally distinct forms of these metabolites. Lipases/esterases and the strongly induced *MAB_1693-MAB_1699* Mce cluster may play a role in releasing and importing, respectively, fatty acyl chains from ECM lipids (including DOPC). Finally, consistent with our proteomics results, we note that several chaperones/chaperonins (GroEL, GroEL1, GroES, etc.) were strongly upregulated in biofilm-grown MABSC, but only during the maturation stage (B5) (Figure 6B and Supplementary Table 3).

Interestingly, in contrast to what had been reported in *M. tuberculosis* and *M. smegmatis* biofilms (Ojha and Hatfull, 2007; Trivedi et al., 2016) but in agreement with observations made on *M. chelonae* biofilms (Vega-Dominguez et al., 2020), two gene clusters related to iron homeostasis (the type VII secretion system ESX-3 and mycobactin biosynthetic genes) were among the most strongly downregulated in our MABSC biofilm model (both B2 vs. P1 and B5 vs. P2) (Figure 6B, Supplementary Figure 4, and Supplementary Table 3).

Despite evidence of an intense remodeling of the cell envelope (affecting arabinogalactan, peptidoglycan, and several as yet undefined pathways involving MmpL-type transporters) during MABSC’s shift to biofilm growth (Supplementary Figure 4 and Supplementary Table 3), genes involved in GPL biosynthesis which we found to be abundant constituents of the ECM of both biofilm and planktonically grown cells (Figure 3 and Table 3) were not found to be differentially expressed. Consistent with their relatively low abundance in the ECM of *Mabs* NJH12 biofilms, genes involved in the biosynthesis of mycolic acids were also not found among the genes expressed at a significantly higher level in early or late biofilms, with the exception of three genes involved in the late stages of their elongation or functionalization (*MAB_3881* encoding a cyclopropane synthase, and *MAB_3897c-MAB_3898c* encoding the HadBC dehydratase). Genes involved in the elongation and assembling of mycolic acids *per se* [e.g., *fadD32 (MAB_0179)*, *pks13 (MAB_0180)*, *inhA (MAB_2722c)*, *acpM (MAB_1878c)*, *kasA (MAB_1877c)*, *kasB (MAB_4608)*, etc.] were, on the contrary, expressed at a significantly lower level in early-stage biofilms and/or late biofilms compared to their planktonic counterparts. This result is in sharp contrast with the situation recently reported for MABSC biofilms formed at the air-liquid interface in minimal Sauton’s medium (Dokic et al., 2021).

RNA-seq results were validated by RT-qPCR on a few selected genes (Supplementary Figure 5).

### Effect of Some Environmental Cues on *Mycobacterium abscessus* Complex Biofilm Formation

**FIGURE 7:**
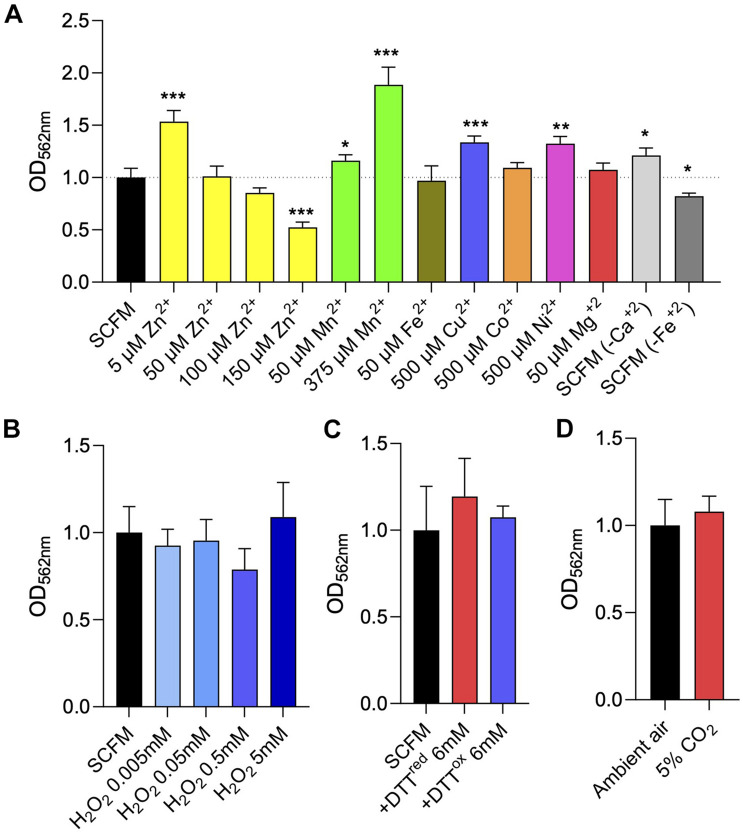
Effect of divalent cation and other environmental cues on MABSC biofilm formation in synthetic CF medium. **(A)** Crystal violet quantification of *Mabs* NJH12 biofilms after 5 days of incubation in standard SCFM medium, SCFM supplemented with various metal ions, or SCFM devoid of CaCl_2_ or FeSO_4_. The growth curves of *Mabs* NJH12 in the corresponding culture media are presented in Supplementary Figure 6. **(B)** Biofilm formation of *Mabs* NJH12 in SCFM in the presence of increasing concentrations of H_2_O_2_. *Mabs* NJH12 biofilm cultures were exposed to 0.005–5 mM H_2_O_2_ from the time of inoculation and biofilm formation was measured by crystal violet staining after 5 days (Geier et al., 2008). **(C)** Impact of thiol reductive stress on *Mabs* NJH12 biofilm formation. *Mabs* NJH12 biofilm cultures were added 6 mM reduced DTT (GoldBio) or 6 mM oxidized DTT (Sigma) at the time of inoculation and incubated for 24 h prior to crystal violet staining. **(D)** Biofilm formation of *Mabs* NJH12 in SCFM under a 5% CO_2_ atmosphere compared to ambient air. Asterisks denote statistically significant differences between enzyme treatment and control groups (standard SCFM medium under ambient air) pursuant to the Student’s *t*-test (*p < 0.05, **p < 0.005, and ***p < 0.0005). Crystal violet values are expressed relative to untreated biofilm values (grown under ambient air) arbitrarily set to 1.

Much like the situation in other bacteria, biofilm formation in mycobacteria is governed by a number of environmental cues including nutrient availability, osmolarity, CO_2_/bicarbonate, metal ions and redox stresses (Carter et al., 2003; Ojha and Hatfull, 2007; Ojha et al., 2008; Rose and Bermudez, 2016; Trivedi et al., 2016; Chakraborty and Kumar, 2019). Bicarbonate was shown to positively influence, in a pH-independent manner, eDNA export in MABSC, *M. chelonae* and *M. avium* (Rose and Bermudez, 2016). *M. avium* biofilm formation on plastic microtiter plates in water, on the other hand, was shown to be dependent on the presence of Ca^2+^, Mg^2+^ and Zn^2+^ (Carter et al., 2003). Zinc is required for biofilm formation in *M. tuberculosis* (Ojha et al., 2008) whereas iron appears to only be required during the late stages of biofilm development in *M. smegmatis* and *M. tuberculosis* (Ojha and Hatfull, 2007; Ojha et al., 2008). Furthermore, the results of our transcriptional profiling studies indicated that magnesium and manganese homeostasis may be important to both the early and maturation stages of MABSC biofilm development (Supplementary Table 3). It was proposed that divalent cations act as stabilizing agents for the negatively charged nucleic acids present in the biofilms (Chakraborty and Kumar, 2019). To determine whether divalent cations impacted the formation of *Mabs* NJH12 biofilms in SCFM, we tested the effect of removing or, on the contrary, supplementing SCFM with Fe^2+^, Mg^2+^, Ca^2+^, Mn^2+^, Cu^2+^, Co^2+^, Ni^2+^, and Zn^2+^. Each ion was tested at the maximum concentration at which it remained soluble in the medium while having no deleterious effect on bacterial planktonic growth and the results are shown in Figure 7A and Supplementary Figure 6. Of the three divalent cations naturally present in SCFM, Fe^2+^, Mg^2+^, and Ca^2+^, only Mg^2+^ was required for MABSC growth in SCFM (Supplementary Figure 6). Omitting CaCl_2_ from the SCFM preparation, or reducing MgCl_2_ to near the minimal concentration required to support normal growth (from the 606 μM normally present in SCFM to 50 μM) had no deleterious impact on biofilm formation whereas omitting FeSO_4_ was slightly inhibitory. Increasing Fe^2+^ concentration to 50 μM, Mn^2+^ concentration to 375 μM, and Cu^2+^, Co^2+^, Ni^2+^ concentrations to 500 μM did not alter the ability of *Mabs* NJH12 to form biofilms. Zn^2+^, however, inhibited biofilm formation in a concentration-dependent manner with an IC_50_ (the concentration of Zn^2+^ required to inhibit 50% of biofilm formation) in the range of ∼ 150 μM (Figure 7A). Thus, of all metals tested, Fe^2+^ slightly promoted MABSC biofilm formation in SCFM whereas Zn^2+^ was inhibitory at relatively high concentrations. Some ions (e.g., Zn^2+^ at 5 μM, Ni^2+^ at 500 μM, Mn^2+^ at 50 and 375 μM and Cu^2+^ at 500 μM) slightly stimulated biofilm development but, for some (Zn^2+^ at 5 μM and Ni^2+^ at 500 μM), this was most likely due to their stimulatory effect on bacterial growth (Supplementary Figure 6).

Because MABSC residing inside necrotizing lung granulomas and in the neutrophil-rich environment of the CF airway is exposed to oxidative stress (Malcolm et al., 2013), and because of previous reports showing that oxidative stress promoted *M. avium* biofilm formation *in vitro* (Geier et al., 2008) and MABSC growth in macrophages (Oberley-Deegan et al., 2010), we next tested the effect of exposing biofilm-grown *Mabs* NJH12 to increasing concentrations of hydrogen peroxide (Geier et al., 2008). Concentrations of H_2_O_2_ up to 5 mM, however, had no significant impact on *Mabs* NJH12 biofilm formation in the SCFM model (Figure 7B). A higher concentration of H_2_O_2_ (50 mM) resulted in the killing of *Mabs* (data not shown). Next, we tested whether thiol reductive stress induced biofilm formation in MABSC as described in *M. tuberculosis* and, more recently, in MABSC grown in minimal Sauton’s medium (Trivedi et al., 2016; Chakraborty et al., 2021). However, again, neither reduced or oxidized DTT (at a final concentration of 6 mM) had any significant effect on the formation of NJH12 biofilms in SCFM (Figure 7C).

Finally, we found that growing *Mabs* NJH12 in SCFM in ambient air or in a 5% CO2 incubator also had no impact on the formation of biofilms (Figure 7D).

## Discussion

Understanding the environmental cues and molecular events leading to surface attachment and generation of a drug-tolerant biofilm has the potential to reveal novel interventions to better control MABSC infections. The studies reported herein highlight the importance of studying MABSC biofilms under conditions that mimic those encountered by the bacterium during host infection since different *in vitro* biofilm models lead to significantly different conclusions with regards to not only the nature of the critical constituents of the ECM but also the way MABSC biofilms respond to environmental cues.

In contrast to MABSC biofilms formed in Sauton’s minimal medium under thiol reductive stress that were readily dispersed by proteinase K (Chakraborty et al., 2021), this enzyme or trypsin had relatively little effect on pre-established *Mabs* NJH12 biofilms, while the reverse was observed with DNase. In our model, DNA was in fact the ECM constituent of bacterial origin whose abundance increased the most (∼10-fold) between planktonic and biofilm-forming conditions. Other enzymes with the ability to disperse pre-established SCFM-grown MABSC biofilms included α-mannosidase, cellulase and phospholipases A1 and A2. eDNA, phospholipids (i.e., exogenous DOPC and endogenous cardiolipin) and polysaccharides are thus key ECM constituents holding clusters of MABSC bacilli together in SCFM, whereas MABSC proteins appeared to play a less important role in this regard despite being abundant constituents of the ECM. It is possible that proteins serve a different primary function in the ECM (e.g., in bacterial metabolism or resistance to stresses) or that they play a more prominent role at other stages of biofilm development such as initial attachment or maturation. Interestingly, the presence of DOPC from the onset of biofilm growth, without impacting growth rate, appeared to slow down the attachment of the bacilli to substratum suggesting that lung surfactant lipids may mitigate the attachment of MABSC biofilms even though, once incorporated into the ECM, they contribute to holding the clusters of bacilli together.

Inter-species and inter-model differences also expressed in the facts that the ECM of SCFM-grown *Mabs* NJH12 contained very different lipids from those reported in *M. chelonae*, *M. tuberculosis*, *M. smegmatis*, and MABSC grown in minimal laboratory media. Indeed, medium-chain fatty acids (palmitic, stearic, palmitoleic and oleic acids) along with cardiolipin, GPLs and SCFM-derived DOPC, rather than free mycolates, dominated the lipid composition of the ECM in the SCFM model. It is possible that some of the free fatty acids found in the ECM originate in the active hydrolysis of DOPC by the bacilli in an effort to recover fatty acyl chains as carbon sources since the level of expression of several lipases/esterase genes significantly increased in biofilm-forming MABSC bacilli relative to planktonically grown cells. To what extent the presence of abundant quantities of lung surfactant phospholipids qualitatively and quantitatively impacts the synthesis and secretion of endogenous lipids, including mycolic acids, by biofilm-forming MABSC during infection is an important question worthy of further investigations. We note, however, that the accumulation of free mycolates in biofilm-forming mycobacteria has to this date solely been reported in bacilli grown in minimal media (M63 or Sauton’s) as pellicles at the liquid-air interface (Ojha et al., 2005, 2008; Ojha and Hatfull, 2007; Vega-Dominguez et al., 2020; Dokic et al., 2021) and could thus be a specific feature associated with these particular types of biofilms.

While RNA-seq analyses revealed similarities between mycobacterial biofilms (e.g., upregulation of oxidoreductase genes and genes involved in isonitrile lipopeptide synthesis, response to hypoxia and redox stress, cell envelope remodeling; lack of induction of GPL biosynthetic genes) (Richards et al., 2019; Vega-Dominguez et al., 2020; Dokic et al., 2021), they also highlighted significant differences between the transcriptional profile of SCFM-grown *Mabs* NJH12 biofilms and those of other mycobacteria, including *Mabs* ATCC 19977, grown in different laboratory media, most likely reflecting inter-species variations as well as differences in the *in vitro* conditions under which the biofilms were formed. Most notable among these differences was the downregulation of genes involved in iron homeostasis in our model (ESX-3 and mycobactin synthesis) and that of mycolic acid biosynthetic genes.

Strikingly, in a rich medium such as SCFM, virtually none of the environmental cues that we tested had any significant impact on the ability of MABSC to form biofilms (Figure 7). This result contrasts with the important effects of varying the ratio of carbon to nitrogen or adding bicarbonate, metal ions or other sources of stress (oxidative stress, thiol reductive stress) on the microaggregation and biofilm-forming capacity of mycobacteria grown in standard laboratory media (Carter et al., 2003; Ojha and Hatfull, 2007; Ojha et al., 2008; Rose and Bermudez, 2016; DePas et al., 2019; Chakraborty et al., 2021; Dokic et al., 2021), and is probably explained by the abundance of nutrients present in SCFM with overlapping functions in biofilm development and the multitude of stress response mechanisms apparently induced in MABSC grown under these conditions. The titration of metal ions and other divalent cations had overall little impact, with the exception of a modestly deleterious effect of omitting FeSO_4_ from the preparation of SCFM. Zinc was the only metal ion found to be inhibitory to biofilm formation, as reported in the case of other bacterial biofilms (Wu et al., 2013), albeit at relatively high concentrations (IC_50_ in the range of 150 μM) that likely precludes its safe therapeutic application.

Collectively, the findings reported herein provide significant new knowledge about the physiological state of MABSC biofilms grown under conditions mimicking the environment of the CF airway. Future studies aimed at determining to what extent the molecular determinants involved in this lifestyle contribute to host colonization, immune evasion and poor treatment outcome could inform novel therapeutic strategies to better control MABSC infections. On the short-term, the finding that eDNA represents a major determinant of MABSC biofilm formation already provides strong support for the use of DNase as an adjunct therapy with the potential to disrupt and reverse the drug tolerance of MABSC biofilms in persons with CF.

## Data Availability Statement

The datasets presented in this study can be found in online repositories. The names of the repository/repositories and accession number(s) can be found below: https://www.ncbi.nlm.nih.gov/bioproject/PRJNA648126.

## Author Contributions

JB, WL, CA, CW, SA, JN, BB, and MJ conceived and designed the experiments, and analyzed the data. VM, ZP, and CK generated the recombinant strains. WL, VJ, SA, MG-J, and ZP prepared biofilm and planktonic cultures, and analyzed biochemically and confocal microscopy imaging the composition of the extracellular matrix. JB, BA, EL, and KM ran biofilm assays under different culture conditions. JB, RD, CW, and CA performed the transcriptional profiling experiments. MJ wrote the manuscript. All authors reviewed and approved the manuscript.

## Conflict of Interest

The authors declare that the research was conducted in the absence of any commercial or financial relationships that could be construed as a potential conflict of interest.

## Publisher’s Note

All claims expressed in this article are solely those of the authors and do not necessarily represent those of their affiliated organizations, or those of the publisher, the editors and the reviewers. Any product that may be evaluated in this article, or claim that may be made by its manufacturer, is not guaranteed or endorsed by the publisher.

## Footnotes

1
https://github.com/hym0405/RNaseH_depletion

## References

[B1] Anders S. Pyl P. T. Huber W. (2015). HTSeq-a Python framework to work with high-throughput sequencing data. *Bioinformatics* 31 166–169. 10.1093/bioinformatics/btu638 25260700 PMC4287950

[B2] Babrak L. Danelishvili L. Rose S. J. Bermudez L. E. (2015a). Microaggregate-associated protein involved in invasion of epithelial cells by *Mycobacterium avium* subsp. *hominissuis*. *Virulence* 6 694–703. 26252358 10.1080/21505594.2015.1072676 PMC4720229

[B3] Babrak L. Danelishvili L. Rose S. J. Kornberg T. Bermudez L. E. (2015b). The environment of “Mycobacterium avium subsp. hominissuis” microaggregates induces synthesis of small proteins associated with efficient infection of respiratory epithelial cells. *Infect. Immun.* 83 625–636. 10.1128/iai.02699-14 25422262 PMC4294266

[B4] Benjamini Y. Hochberg Y. (2000). On the adaptive control of the false discovery fate in multiple testing with independent statistics. *J. Educ. Behav. Stat.* 25 60–83. 10.2307/1165312

[B5] Bharti S. Maurya R. K. Venugopal U. Singh R. Akhtar M. S. Krishnan M. Y. (2020). Rv1717 is a cell wall - associated beta-galactosidase of *Mycobacterium tuberculosis* that is involved in biofilm dispersion. *Front. Microbiol.* 11:611122. 10.3389/fmicb.2020.611122 33584576 PMC7873859

[B6] Bhatt K. Machado H. Osorio N. S. Sousa J. Cardoso F. Magalhaes C. (2018). A nonribosomal peptide synthase gene driving virulence in *Mycobacterium tuberculosis*. *mSphere* 3:e00352-18. 10.1128/mSphere.00352-18 30381350 PMC6211224

[B7] Carter G. Wu M. Drummond D. C. Bermudez L. E. (2003). Characterization of biofilm formation by clinical isolates of *Mycobacterium avium*. *J. Med. Microbiol.* 52 747–752. 10.1099/jmm.0.05224-0 12909649

[B8] Chakraborty P. Bajeli S. Kaushal D. Radotra B. D. Kumar A. (2021). Biofilm formation in the lung contributes to virulence and drug tolerance of *Mycobacterium tuberculosis*. *Nat. Commun.* 12:1606. 10.1038/s41467-021-21748-6 PMC7952908 33707445

[B9] Chakraborty P. Kumar A. (2019). The extracellular matrix of mycobacterial biofilms: could we shorten the treatment of mycobacterial infections? *Microb. Cell* 6 105–122. 10.15698/mic2019.02.667 30740456 PMC6364259

[B10] Chan K. Knaak T. Satkamp L. Humbert O. Falkow S. Ramakrishnan L. (2002). Complex pattern of *Mycobacterium marinum* gene expression during long-term granulomatous infection. *Proc. Natl. Acad. Sci. U.S.A.* 99 3920–3925. 10.1073/pnas.002024599 11891270 PMC122624

[B11] Clary G. Sasindran S. J. Nesbitt N. Mason L. Cole S. Azad A. (2018). *Mycobacterium abscessus* smooth and rough morphotypes form antimicrobial-tolerant biofilm phenotypes but are killed by acetic acid. *Antimicrob. Agents Chemother.* 62:e01782-17. 10.1128/AAC.01782-17 PMC5826145 29311080

[B12] Davidson L. B. Nessar R. Kempaiah P. Perkins D. J. Byrd T. F. (2011). Mycobacterium abscessus glycopeptidolipid prevents respiratory epithelial TLR2 signaling as measured by HbetaD2 gene expression and IL-8 release. *PLoS One* 6:e29148. 10.1371/journal.pone.0029148 22216191 PMC3244437

[B13] DePas W. H. Bergkessel M. Newman D. K. (2019). Aggregation of nontuberculous mycobacteria is regulated by carbon-nitrogen balance. *mBio* 10:e01715-9. 10.1128/mBio.01715-19 31409683 PMC6692514

[B14] Dhar N. McKinney J. D. (2010). Mycobacterium tuberculosis persistence mutants identified by screening in isoniazid-treated mice. *Proc. Natl. Acad. Sci. U.S.A.* 107 12275–12280. 10.1073/pnas.1003219107 20566858 PMC2901468

[B15] Dokic A. Peterson E. Arrieta-Ortiz M. L. Pan M. Di Maio A. Baliga N. (2021). *Mycobacterium abscessus* biofilms produce an extracellular matrix and have a distinct mycolic acid profile. *Cell Surf.* 7:100051. 10.1016/j.tcsw.2021.100051 33912773 PMC8066798

[B16] Faria S. Joao I. Jordao L. (2015). General overview on nontuberculous mycobacteria, biofilms, and human infection. *J. Pathog.* 2015:809014. 10.1155/2015/809014 PMC4649093 26618006

[B17] Fennelly K. P. Ojano-Dirain C. Yang Q. Liu L. Lu L. Progulske-Fox A. (2016). Biofilm formation by *Mycobacterium abscessus* in a lung cavity. *Am. J. Respir. Crit. Care Med.* 193 692–693. 26731090 10.1164/rccm.201508-1586IM

[B18] Floto R. A. Haworth C. S. (2015). The growing threat of nontuberculous mycobacteria in CF. *J. Cyst. Fibros* 14 1–2. 10.1016/j.jcf.2014.12.002 25487786

[B19] Garcia-Perez B. E. Villagomez-Palatto D. A. Castaneda-Sanchez J. I. Coral-Vazquez R. M. Ramirez-Sanchez I. Ordonez-Razo R. M. (2011). Innate response of human endothelial cells infected with mycobacteria. *Immunobiology* 216 925–935. 10.1016/j.imbio.2011.01.004 21397978

[B20] Geier H. Mostowy S. Cangelosi G. A. Behr M. A. Ford T. E. (2008). Autoinducer-2 triggers the oxidative stress response in *Mycobacterium avium*, leading to biofilm formation. *Appl. Environ. Microbiol.* 74 1798–1804. 10.1128/aem.02066-07 18245256 PMC2268301

[B21] Gerasimova A. Kazakov A. E. Arkin A. P. Dubchak I. Gelfand M. S. (2011). Comparative genomics of the dormancy regulons in mycobacteria. *J. Bacteriol.* 193 3446–3452. 10.1128/jb.00179-11 21602344 PMC3133309

[B22] Harris N. C. Sato M. Herman N. A. Twigg F. Cai W. Liu J. (2017). Biosynthesis of isonitrile lipopeptides by conserved nonribosomal peptide synthetase gene clusters in Actinobacteria. *Proc. Natl. Acad. Sci. U.S.A.* 114 7025–7030. 10.1073/pnas.1705016114 28634299 PMC5502637

[B23] Hoiby N. (2017). A short history of microbial biofilms and biofilm infections. *APMIS* 125 272–275. 10.1111/apm.12686 28407426

[B24] Hotter G. S. Wards B. J. Mouat P. Besra G. S. Gomes J. Singh M. (2005). Transposon mutagenesis of Mb0100 at the ppe1-nrp locus in *Mycobacterium bovis* disrupts phthiocerol dimycocerosate (PDIM) and glycosylphenol-PDIM biosynthesis, producing an avirulent strain with vaccine properties at least equal to those of M. *bovis BCG*. *J. Bacteriol.* 187 2267–2277. 10.1128/jb.187.7.2267-2277.2005 15774869 PMC1065232

[B25] Huang D. W. Sherman B. T. Lempicki R. A. (2009). Systematic and integrative analysis of large gene lists using DAVID bioinformatics resources. *Nat. Protoc.* 4 44–57. 10.1038/nprot.2008.211 19131956

[B26] Huang Y. Sheth R. U. Kaufman A. Wang H. H. (2020). Scalable and cost-effective ribonuclease-based rRNA depletion for transcriptomics. *Nucleic Acids Res.* 48:e20. 10.1093/nar/gkz1169 31879761 PMC7038938

[B27] Jiang H. Lei R. Ding S. W. Zhu S. (2014). Skewer: a fast and accurate adapter trimmer for next-generation sequencing paired-end reads. *BMC Bioinform.* 15:182. 10.1186/1471-2105-15-182 24925680 PMC4074385

[B28] Kaur D. McNeil M. R. Khoo K. H. Chatterjee D. Crick D. C. Jackson M. (2007). New insights into the biosynthesis of mycobacterial lipomannan arising from deletion of a conserved gene. *J. Biol. Chem.* 282 27133–27140. 10.1074/jbc.m703389200 17606615

[B29] Langmead B. Salzberg S. L. (2012). Fast gapped-read alignment with Bowtie 2. *Nat. Methods* 9 357–359. 10.1038/nmeth.1923 22388286 PMC3322381

[B30] Love M. I Huber W. Anders S. (2014). Moderated estimation of fold change and dispersion for RNA-seq data with DESeq2. *Genome Biol.* 15:550. 10.1186/s13059-014-0550-8 PMC4302049 25516281

[B31] Malcolm K. C. Nichols E. M. Caceres S. M. Kret J. E. Martiniano S. L. Sagel S. D. (2013). *Mycobacterium abscessus* induces a limited pattern of neutrophil activation that promotes pathogen survival. *PLoS One* 8:e57402. 10.1371/journal.pone.0057402 23451220 PMC3581440

[B32] Marshall N. J. Goodwin C. J. Holt S. J. (1995). A critical assessment of the use of microculture tetrazolium assays to measure cell growth and function. *Growth Regul.* 5 69–84. 7627094

[B33] Marsollier L. Brodin P. Jackson M. Kordulakova J. Tafelmeyer P. Carbonnelle E. (2007). Impact of *Mycobacterium ulcerans* biofilm on transmissibility to ecological niches and Buruli ulcer pathogenesis. *PLoS Pathog.* 3:e62. 10.1371/journal.ppat.0030062 17480118 PMC1864991

[B34] Martin A. Camacho M. Portaels F. Palomino J.-C. (2003). Resazurin microtiter assay plate testing of *Mycobacterium tuberculosis* susceptibilities to second-line drugs: rapid, simple, and inexpensive method. *Antimicrob. Agents Chemother.* 47 3616–3619. 14576129 10.1128/AAC.47.11.3616-3619.2003 PMC253784

[B35] Martiniano S. L. Nick J. A. Daley C. L. (2019). Nontuberculous mycobacterial infections in cystic fibrosis. *Thorac. Surg. Clin.* 29 95–108. 30454926 10.1016/j.thorsurg.2018.09.008

[B36] Matsuyama M. Martins A. J. Shallom S. Kamenyeva O. Kashyap A. Sampaio E. P. (2018). Transcriptional response of respiratory epithelium to nontuberculous mycobacteria. *Am. J. Respir. Cell Mol. Biol.* 58 241–252. 28915071 10.1165/rcmb.2017-0218OC PMC5806000

[B37] Miranda-CasoLuengo A. A. Staunton P. M. Dinan A. M. Lohan A. J. Loftus B. J. (2016). Functional characterization of the *Mycobacterium abscessus* genome coupled with condition specific transcriptomics reveals conserved molecular strategies for host adaptation and persistence. *BMC Genomics* 17:553. 10.1186/s12864-016-2868-y 27495169 PMC4974804

[B38] Nett J. E. Cain M. T. Crawford K. Andes D. R. (2011). Optimizing a Candida biofilm microtiter plate model for measurement of antifungal susceptibility by tetrazolium salt assay. *J. Clin. Microbiol.* 49 1426–1433. 10.1128/JCM.02273-10 21227984 PMC3122839

[B39] Oberley-Deegan R. E. Rebits B. W. Weaver M. R. Tollefson A. K. Bai X. McGibney M. (2010). An oxidative environment promotes growth of *Mycobacterium abscessus*. *Free Radic. Biol. Med.* 49 1666–1673. 10.1016/j.freeradbiomed.2010.08.026 20807564 PMC2970643

[B40] Ojha A. Anand M. Bhatt A. Kremer L. Jacobs W. R. Jr. (2005). GroEL1: a dedicated chaperone involved in mycolic acid biosynthesis during biofilm formation in mycobacteria. *Cell* 123 861–873. 10.1016/j.cell.2005.09.012 16325580

[B41] Ojha A. Hatfull G. F. (2007). The role of iron in *Mycobacterium smegmatis* biofilm formation: the exochelin siderophore is essential in limiting iron conditions for biofilm formation but not for planktonic growth. *Mol. Microbiol.* 66 468–483. 10.1111/j.1365-2958.2007.05935.x 17854402 PMC2170428

[B42] Ojha A. K. Baughn A. D. Sambandan D. Hsu T. Trivelli X. Guerardel Y. (2008). Growth of *Mycobacterium tuberculosis* biofilms containing free mycolic acids and harbouring drug-tolerant bacteria. *Mol. Microbiol.* 69 164–174. 10.1111/j.1365-2958.2008.06274.x 18466296 PMC2615189

[B43] Ortalo-Magné A. Dupont M. A. Lemassu A. Andersen A. B. Gounon P. Daffé M. (1995). Molecular composition of the outermost capsular material of the tubercle bacillus. *Microbiology* 141(Pt 7), 1609–1620. 10.1099/13500872-141-7-1609 7551029

[B44] Park H. D. Guinn K. M. Harrell M. I. Liao R. Voskuil M. I. Tompa M. (2003). Rv3133c/dosR is a transcription factor that mediates the hypoxic response of *Mycobacterium tuberculosis*. *Mol. Microbiol.* 48 833–843. 10.1046/j.1365-2958.2003.03474.x 12694625 PMC1992516

[B45] Park I. K. Olivier K. N. (2015). Nontuberculous mycobacteria in cystic fibrosis and non-cystic fibrosis bronchiectasis. *Semin. Respir. Crit. Care Med.* 36 217–224. 10.1055/s-0035-1546751 25826589 PMC7171444

[B46] Pearson W. R. (2013). Selecting the right similarity-scoring matrix. *Curr. Protoc. Bioinform.* 43 351–359. 10.1002/0471250953.bi0305s43 24509512 PMC3848038

[B47] Qvist T. Eickhardt S. Kragh K. N. Andersen C. B. Iversen M. Hoiby N. (2015). Chronic pulmonary disease with *Mycobacterium abscessus* complex is a biofilm infection. *Eur. Respir. J.* 46 1823–1826. 10.1183/13993003.01102-2015 26493807

[B48] Richards J. P. Cai W. Zill N. A. Zhang W. Ojha A. K. (2019). Adaptation of *Mycobacterium tuberculosis* to biofilm growth is genetically linked to drug tolerance. *Antimicrob. Agents Chemother.* 63:e01213-9. 10.1128/AAC.01213-19 31501144 PMC6811442

[B49] Richards J. P. Ojha A. K. (2014). Mycobacterial biofilms. *Microbiol. Spectr.* 2:MGM2-0004-2013. 10.1128/microbiolspec.MGM2-0004-2013 26104368

[B50] Rose S. J. Babrak L. M. Bermudez L. E. (2015). *Mycobacterium avium* possesses extracellular DNA that contributes to biofilm formation, structural integrity, and tolerance to antibiotics. *PLoS One* 10:e0128772. 10.1371/journal.pone.0128772 26010725 PMC4444313

[B51] Rose S. J. Bermudez L. E. (2014). *Mycobacterium avium* biofilm attenuates mononuclear phagocyte function by triggering hyperstimulation and apoptosis during early infection. *Infect. Immun.* 82 405–412. 24191301 10.1128/IAI.00820-13 PMC3911830

[B52] Rose S. J. Bermudez L. E. (2016). Identification of bicarbonate as a trigger and genes involved with extracellular DNA export in mycobacterial biofilms. *mBio* 7:e01597-16. 10.1128/mBio.01597-16 27923918 PMC5142616

[B53] Rossi E. La Rosa R. Bartell J. A. Marvig R. L. Haagensen J. A. J. Sommer L. M. (2021). *Pseudomonas aeruginosa* adaptation and evolution in patients with cystic fibrosis. *Nat. Rev. Microbiol.* 19 331–342. 33214718 10.1038/s41579-020-00477-5

[B54] Sartain M. J. Dick D. L. Rithner C. D. Crick D. C. Belisle J. T. (2011). Lipidomic analyses of *Mycobacterium tuberculosis* based on accurate mass measurements and the novel Mtb LipidDB. *J. Lipid Res.* 52 861–872. 21285232 10.1194/jlr.M010363 PMC3073466

[B55] Trivedi A. Mavi P. S. Bhatt D. Kumar A. (2016). Thiol reductive stress induces cellulose-anchored biofilm formation in *Mycobacterium tuberculosis*. *Nat. Commun.* 7:11392. 10.1038/ncomms11392 27109928 PMC4848537

[B56] Turner K. H. Wessel A. K. Palmer G. C. Murray J. L. Whiteley M. (2015). Essential genome of *Pseudomonas aeruginosa* in cystic fibrosis sputum. *Proc. Natl. Acad. Sci. U.S.A.* 112 4110–4115. 10.1073/pnas.1419677112 25775563 PMC4386324

[B57] Vega-Dominguez P. Peterson E. Pan M. Di Maio A. Singh S. Umapathy S. (2020). Biofilms of the non-tuberculous Mycobacterium chelonae form an extracellular matrix and display distinct expression patterns. *Cell Surf.* 6:100043. 10.1016/j.tcsw.2020.100043 32803022 PMC7421604

[B58] Viljoen A. Herrmann J. L. Onajole O. K. Stec J. Kozikowski A. P. Kremer L. (2017). Controlling extra- and intramacrophagic *Mycobacterium abscessus* by targeting mycolic acid transport. *Front. Cell. Infect. Microbiol.* 7:388. 10.3389/fcimb.2017.00388 28920054 PMC5585149

[B59] Wiersma C. J. Belardinelli J. M. Avanzi C. Angala S. K. Everall I. Angala B. (2020). Cell surface remodeling of *Mycobacterium abscessus* under cystic fibrosis airway growth conditions. *ACS Infect. Dis.* 6 2143–2154. 10.1021/acsinfecdis.0c00214 32551551

[B60] Wu C. Labrie J. Tremblay Y. D. Haine D. Mourez M. Jacques M. (2013). Zinc as an agent for the prevention of biofilm formation by pathogenic bacteria. *J. Appl. Microbiol.* 115 30–40. 23509865 10.1111/jam.12197

[B61] Xiang X. Deng W. Liu M. Xie J. (2014). Mycobacterium biofilms: factors involved in development, dispersal, and therapeutic strategies against biofilm-relevant pathogens. *Crit. Rev. Eukaryot. Gene Expr.* 24 269–279. 10.1615/CritRevEukaryotGeneExpr.2014010545 25072151

[B62] Yamazaki Y. Danelishvili L. Wu M. Hidaka E. Katsuyama T. Stang B. (2006). The ability to form biofilm influences *Mycobacterium avium* invasion and translocation of bronchial epithelial cells. *Cell Microbiol.* 8 806–814. 10.1111/j.1462-5822.2005.00667.x 16611229

