# Supplementary material for: Unique Features of Mycobacterium abscessus Biofilms Formed in Synthetic Cystic Fibrosis Medium
Source: Front Microbiol. 2021 Oct 29;12:743126. doi: 10.3389/fmicb.2021.743126 (PMC8586431; doi:10.3389/fmicb.2021.743126)
Supplement: Supplementary file 1 [file Data_Sheet_1.pdf]

Supplementary Material

Supplementary Figures

Figure S1: Planktonic growth of *Mabs* NJH12 in SCFM medium.
The SCFM medium used herein is identical to that used in the biofilm assays (black line). The green line shows the growth of *Mabs* NJH12 in the same SCFM medium devoid of DOPC. The clinical isolate shows increased aggregation in this medium initially but grows as a well-dispersed culture after 24 hours. The results shown are averages ± standard deviations of three biological replicates.

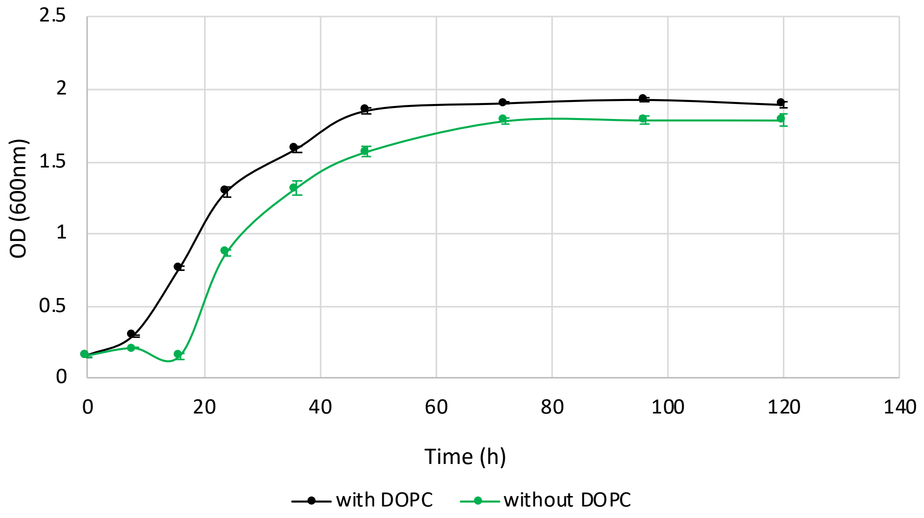

<u>Figure S2</u>: Protein profiles of extracellular matrices prepared from planktonically-grown and biofilm-grown *Mabs* NJH12.

SDS-PAGE analysis of proteins from (1) total cell lysates prepared from 5-days old *Mabs* NJH12 biofilm-grown cells, (2) the ECM of 5-days old *Mabs* NJH12 biofilm-grown cells, and (3) the ECM of 2-days-old *Mabs* NJH12 planktonic-grown cells. 250 ng of protein were loaded per lane. Proteins were revealed by Coomassie blue stain.

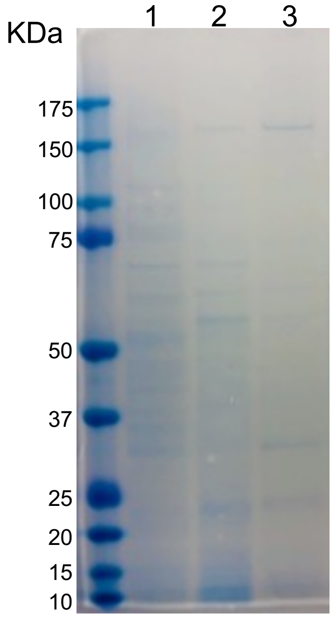

<u>Figure S3</u>: MABSC biofilm formation in SCFM devoid of DOPC.

Mabs NJH12 biofilm formation in medium devoid of DOPC (white bars) versus regular SCFM medium (black bars) as monitored by Crystal violet staining (A) and CFU counting (B). Shown are averages ± standard deviations of triplicate wells. (C) Development of mCHERRY-expressing *Mabs* NJH12 biofilms in medium devoid of DOPC as monitored by fluorescence confocal imaging. 2D (left panels) and 3D (right panels) views of the biofilms are shown. Biofilms strongly attach to the substratum as of day 2 and their density increases over time until they reach maturation on day 4 or 5 and start detaching from the substratum on day 6.

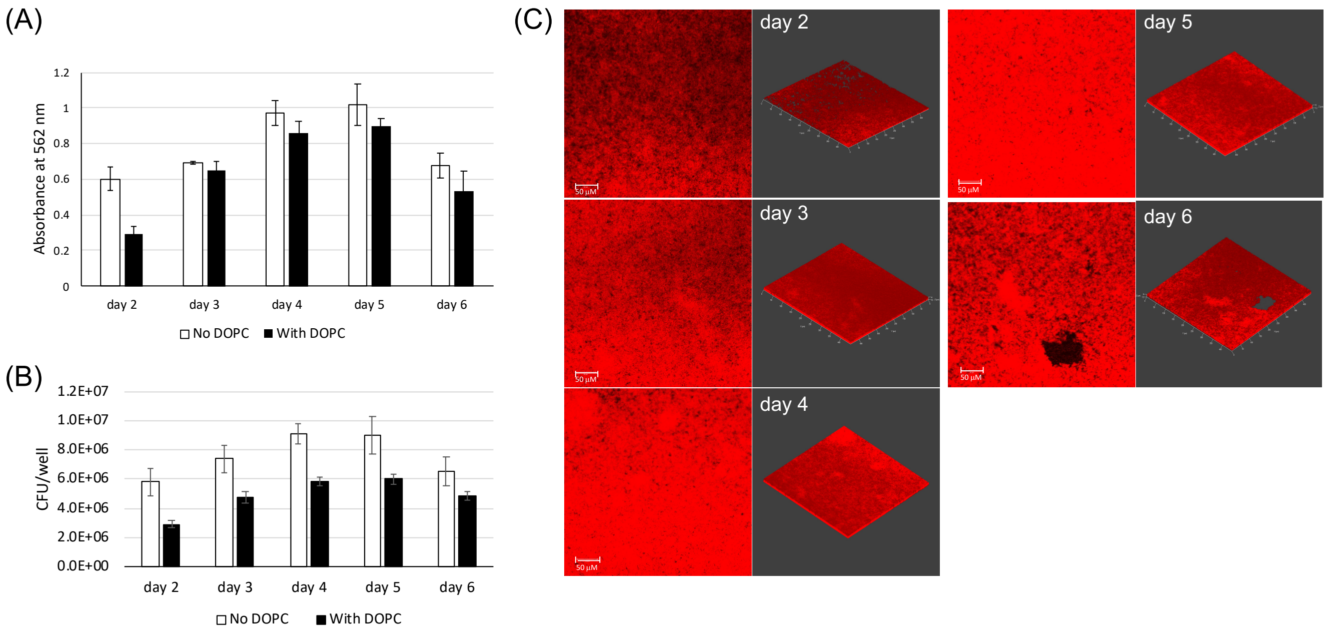

<u>Figure S4</u>: Enriched metabolic pathways in *Mabs* NJH12 biofilms.
Lists of enriched metabolic pathways showing a FDR< 0.05 and fold-enrichment >2 changes deciphered from the set of up-regulated (UP, green) and down-regulated (DWN, red) genes in the different comparisons: B2 vs. P1, B5 vs. P2 and B2 vs. P1 and B5 vs. P2. The x-axis represents the fold enrichment (indicating the magnitude of enrichment in our dataset against the population background based on analysis using DAVID bioinformatics tools). The y-axis shows the pathway categories. The quantity of DE genes associated with each functional term is indicated by the size of each circle, and each circle is color-coded using an FDR-based color gradient. The figures were drawn in R using the ggplot2 package based on the data showed in Table S4. There was no enrichment for the down-regulated genes in B5 vs. P2, or for the up-regulated genes in B2 vs. P1 and B5 vs. P2.

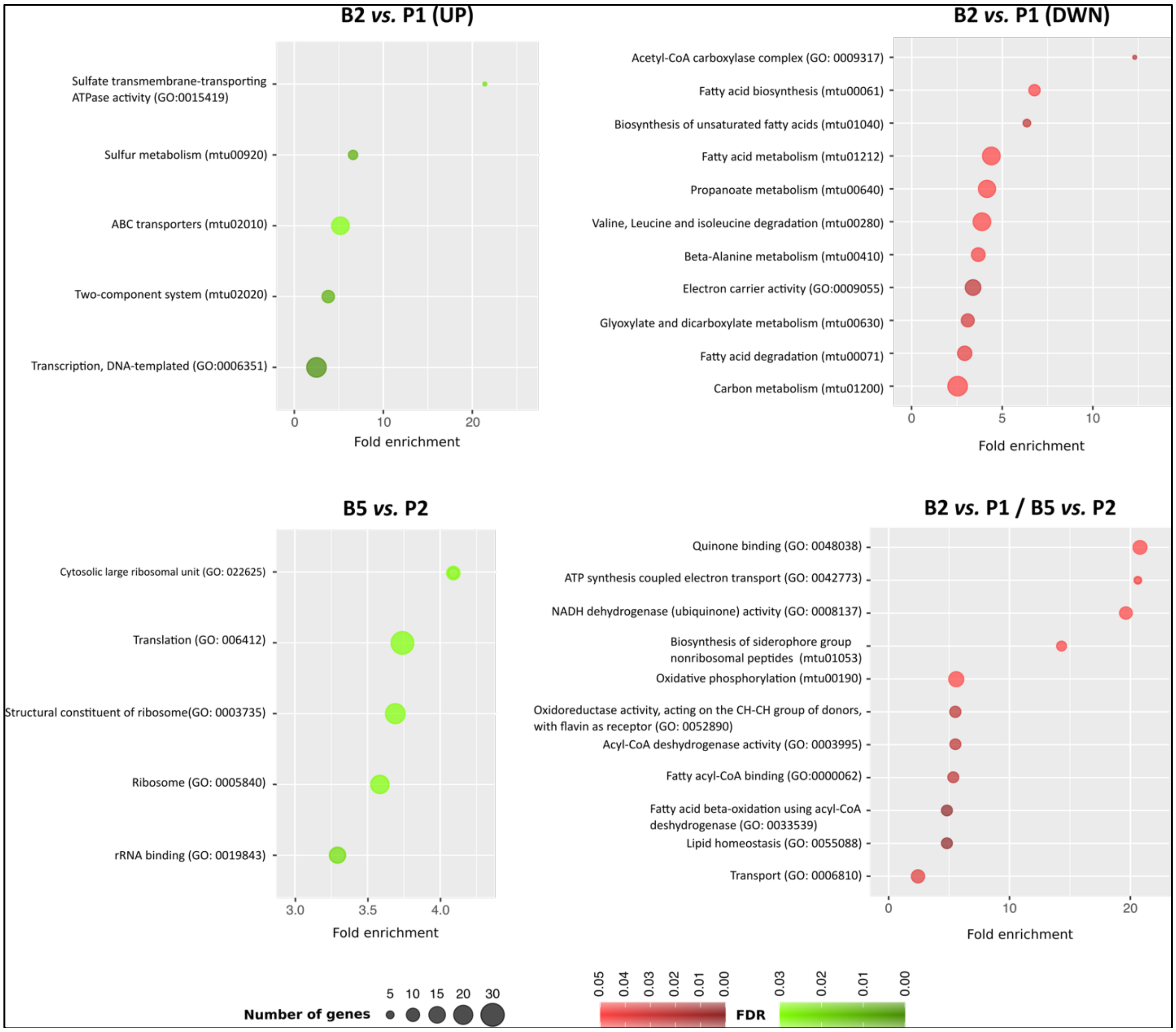

Functional enrichment using DAVID showed that 5 and 11 pathways were enriched in the up- and down-regulated genes, respectively, in early-stage biofilms (B2 *vs.* P1, Table S4), while 5 pathways (mtu03010 is the same as GO:0005840 and thus was only counted once) were significantly enriched in the up-regulated genes of late-phase (mature) biofilms (B5 *vs.* P2, no enrichment in the down-regulated genes). We finally identified enriched metabolic pathways

specific to biofilms in general by pooling DE genes found in both comparisons. Significantly enriched pathways (n = 11) were only found in the down-regulated gene set [Table S4].

See the results section for details about some specific pathways. Other significantly DE genes between biofilm-forming and planktonically-grown cells (B2 vs P1 and/or B5 vs P2) shown here largely consist in genes involved in amino acid import and metabolism (many of which were previously reported to be expressed at high levels in the amino acid-rich SCFM2 [Wiersma *et al.,* 2020; *ACS Infect. Dis*. **6**, 2143-2154]), fatty acid/lipid/carbon metabolism, DNA replication, transcription, and protein synthesis [Table S3; Table S4]. These differences in gene expression are likely reflective of differences in replication rates and metabolism between early-stage biofilm or planktonic cultures and late-stage and stationary planktonic cultures.

<u>Figure S5</u>: RT-qPCR validation of RNAseq data.
RNA-seq (normalized gene counts and log_2 fold-change) (black bars) and quantitative reverse transcription-PCR (RT-qPCR) (red bars) show six DE genes in developing (B2) or mature (B5) biofilms relative to early (P1) and late (P2) log phase planktonic cultures. Ratios of *genes*/*sigA* mRNA are means ± standard deviations (n = 3 RNA extractions and RT-qPCR reactions).

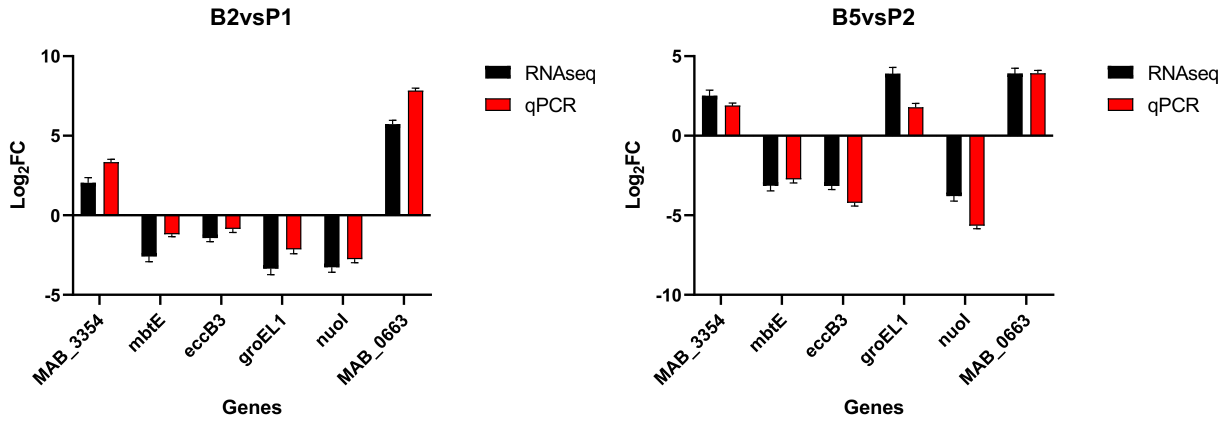

<u>Figure S6</u>: Impact of divalent cations on *Mabs* NJH12 growth in synthetic CF medium. Complete medium refers to SCFM. Complete medium normally contains 3.6 µM $FeSO_4$, 606 µM $MgCl_2$ and 1754 µM $CaCl_2$. Precipitation was observed for $MnSO_4$ above 375 µM and for $FeSO_4$ above 50 µM. $CuSO_4$, $NiCl_2$ and $CoCl_2$ inhibited growth at the highest concentration tested (5 mM) while $ZnSO_4$ started having a slightly deleterious effect on growth at concentrations ≥ 150 µM.

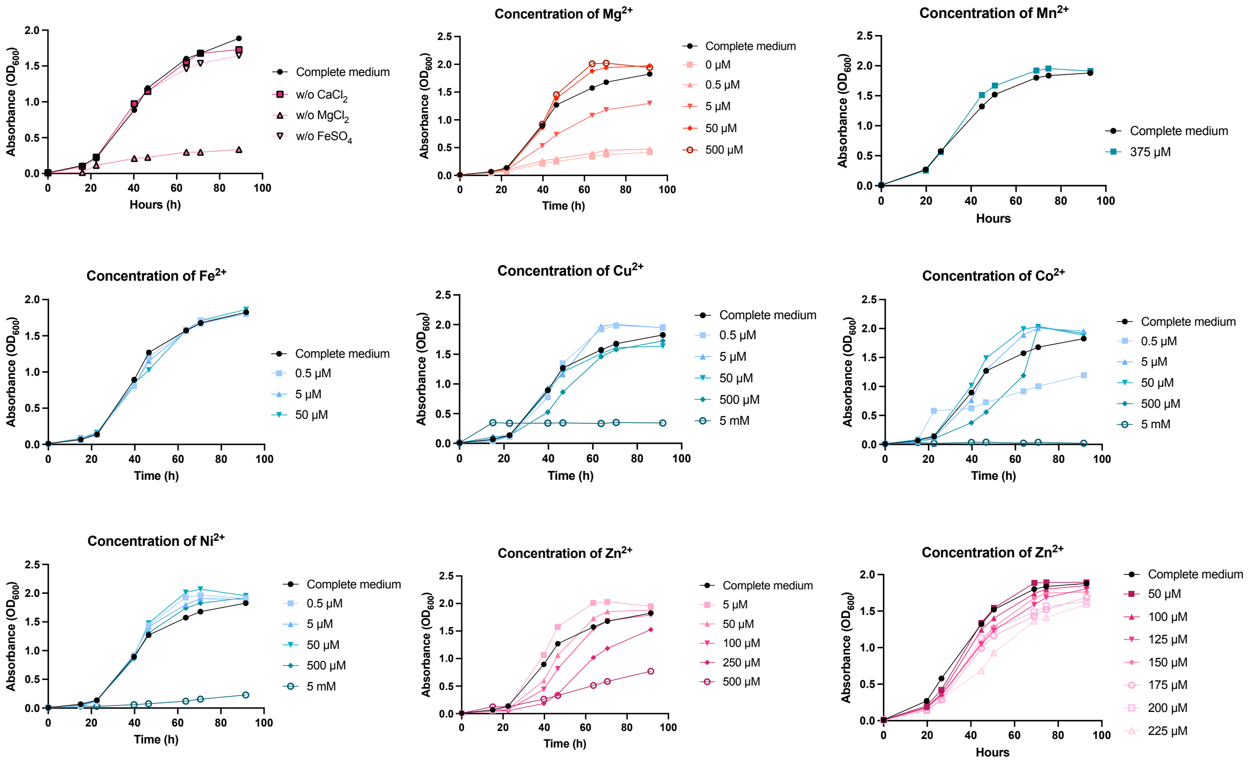

Supplementary Tables

Table S1 [see separate Excel file]: ssDNA oligo probes covering *Mabs* 16s and 23s rRNA used in rRNA depletion.

Table S2 [see separate Excel file]: ECM proteins from planktonic and biofilm-grown *Mabs* NJH12.
A: Unique ECM biofilm proteins (from day 5 biofilms; not found in the ECM of planktonically-grown cells after two and five days). Proteins highlighted in orange are expected to be under control of the DosRS two-component system regulator.
B: Proteins in significantly greater abundance in mature biofilm ECM (day 5) vs ECM from planktonically-grown bacilli (collected after two days of growth; see growth curve in Fig. S1). Two biological replicates were analyzed for each culture conditions and differences between biofilm and planktonic conditions analyzed using a paired Student *t*-test ($p < 0.01$ or 0.05). 75 proteins were identified as significantly increased in the ECM of biofilm-grown cultures (with $p < 0.05$).
In A. and B., asterisks denote proteins whose encoding genes were found to be expressed at significantly higher levels under biofilm growth conditions (see Table S3).

Table S3 [see separate Excel file]: RNA-seq dataset of differentially expressed genes in all comparisons.
Differentially expressed (DE) genes in *Mabs* NJH12 cultured planktonically for 1 day (P1) vs as a biofilm for 2 days (B2), or planktonically for 2 days (P2) vs as a biofilm for 5 days (B5) were defined as ≥ 2.0 or ≤- 2.0-fold changes in expression with a false discovery rate adjusted *p*-value < 0.05.

Table S4 [see separate Excel file]: Results of gene ontology analyses for DE genes (upregulated genes in green; downregulated genes in red) in B2 vs P1 (A), DE genes in B5 vs P2 (B), and DE genes common to both comparisons (C) (DAVID; FDR < 0.05, FE >2).
